# Cerebellum-inspired neural network of supervised learning with tensor-based sparse coding for multi-class classification

**DOI:** 10.1371/journal.pcbi.1014595

**Published:** 2026-08-18

**Authors:** Runguang Zhou, Douglas Zhou, Songting Li, Xiaoyu Chen

**Affiliations:** 1 School of Mathematical Sciences, Shanghai Jiao Tong University, Shanghai, China; 2 Institute of Natural Sciences, Shanghai Jiao Tong University, Shanghai, China; 3 Ministry of Education Key Laboratory of Scientific and Engineering Computing, Shanghai Jiao Tong University, Shanghai, China; 4 Shanghai Frontier Science Center of Modern Analysis, Shanghai Jiao Tong University, Shanghai, China; 5 State Key Laboratory of Synergistic Chem-Bio Synthesis, Shanghai Jiao Tong University, Shanghai, China; Shanghai Institute of Nutrition and Health, Chinese Academy of Sciences, CHINA

## Abstract

Under the Marr-Ito-Albus framework, the cerebellum performs supervised learning in Purkinje cells upon the unsupervised sparse representations generated within granule cells, contributing fundamentally to associative learning in motor control. However, the specific mechanisms through which cerebellar circuitry and plasticity rules enable supervised learning, and properties of the sparse coding induced by cerebellar architectural constraints, remain poorly characterized. To address this, we first established a sparse coding mechanism inspired by anatomical and physiological properties of the granular layer, including input-sharing connectivity from mossy fibers, Golgi-cell-mediated localized feedback inhibition for winner-take-all sparsification, and activity-dependent bias adjustments. This sparse coding, implemented via efficient tensor-based computations, preserves local neighborhood structures as revealed in a geometric interpretation. Furthermore, we demonstrated that error signals transmitted via climbing fibers to Purkinje cells, integrated with intrinsic plasticity, drive efficient learning of the sparse representations for multi-class classification, leading to accurate population coding for judgments in the cerebellar nuclei. The resultant cerebellum-inspired neural network model achieved a test accuracy of 90.50±0.10% on Fashion-MNIST, performance comparable to a backpropagation-trained single-hidden-layer feedforward neural network, while providing more than 5× computational acceleration. The model further exhibited proof-of-principle closed-loop control capability in simplified motor tasks, including balancing a cart-attached pole and controlling a robotic arm to reach targets. Our study delineates cerebellum-inspired sparse coding and supervised learning mechanisms, and demonstrates robust performance of the cerebellum-inspired neural network across multiple task domains. These results suggest that cerebellum-inspired architectures would provide useful design principles for lightweight neural networks in resource-limited and latency-critical applications.

## Author summary

We established a cerebellum-inspired computational framework incorporating random-feature-based sparse coding and climbing-fiber-driven supervised learning mechanisms. The granular-layer-inspired architecture, combined with an unsupervised learning technique, exhibits robust feature representation capabilities, enabling performance comparable to fully supervised three-layer artificial neural networks trained via backpropagation, while providing substantial computational acceleration. By integrating intrinsic plasticity and population coding, the Purkinje cells and the cerebellar nuclei are modeled as a linear multi-class classifier trained with instructive error signals from the climbing fibers, which demonstrates high learning efficiency and stability when processing sparse representations from the upstream granular layer. Implemented using tensor-based operations, the cerebellum-inspired neural network demonstrates proof-of-principle supervised learning capabilities in observation-action mapping, suggesting potential applications as a lightweight module in dynamic control systems.

## 1 Introduction

The Marr-Ito-Albus framework, a cornerstone of computational neuroscience theory, establishes the cerebellum as a supervised learning system [1–4]. Within this framework, associative learning, particularly eyeblink conditioning in response to paired visual cues and air-puff punishments, is the most extensively studied experimental paradigm among the diverse functions of motor adaptations in the cerebellum [5,6]. As is well-documented, climbing fibers (CFs) from the inferior olive (IO) transmit error signals that induce complex spikes in Purkinje cells (PCs), triggering long-term depression (LTD) at parallel fiber (PF) synapses, which mediates disinhibition of downstream cerebellar nuclei (CN) neurons to generate the adaptive eyeblink response and minimize exposure to the instructive air-puff punishment [7].

**Fig 1:**
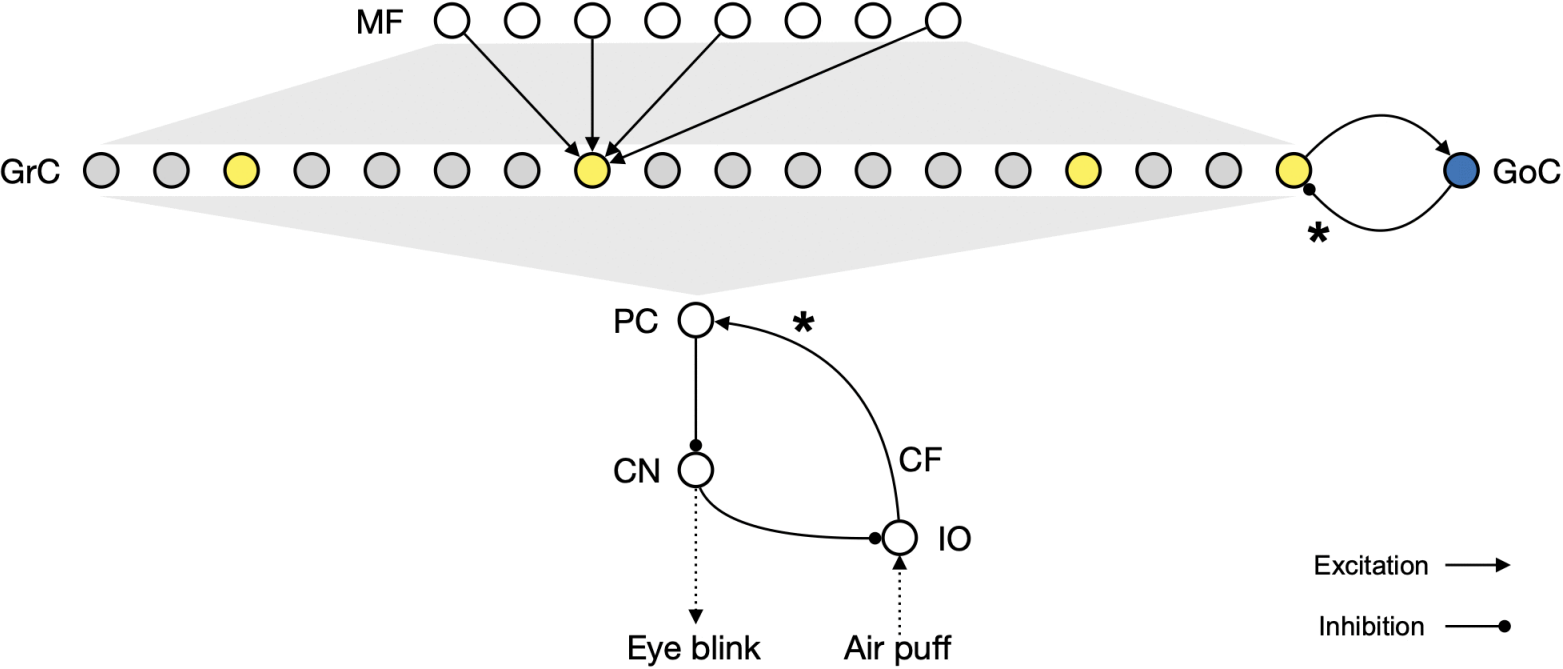
Supervised learning framework of the cerebellum. The CF-error-driven supervised learning mechanisms, together with properties of GrC sparse representations (indicated by asterisks), require further characterization. Yellow nodes indicate the winners in GrCs.

Despite the elegance of the classic model, where PF-PC synaptic depression aligns with the inhibitory nature of PCs, several issues remain unresolved (Fig 1) [8]. As noted by Albus, the unidirectional nature of LTD constitutes an inherently inefficient learning mechanism. Furthermore, the CFs have long been characterized as transmitting “all-or-none” error signals to PCs, imposing significant limitations on cerebellar learning [9]. Such binary signaling could only indicate between correct and incorrect responses, failing to encode crucial distinctions between two fundamentally different error types, i.e., false negative (FN) and false positive (FP) errors [10].

Recent efforts to address this limitation have focused on straightforwardly introducing graded error signals through multiple firing patterns [11], or incorporating information about PC firing state via calcium-dependent plasticity mechanisms [12]. However, Kim et al. [13] have demonstrated that optogenetic inhibition of the IO, which suppresses CF firings, is sufficient to induce extinction in eyeblink conditioning, i.e., learning not responding to previously conditioned cues when the instructive air-puff is no longer presented, suggesting a different coding strategy for errors indirectly provided by CF firing activities. These findings highlight the pressing need to characterize specific mechanisms through which cerebellar circuitry and plasticity rules enable efficient supervised learning.

Furthermore, recent studies have demonstrated that the upstream circuitry involves a massively expanded population of roughly 50 billion granule cells (GrCs), which form an unsupervised feature-representation layer processing mossy fiber (MF) sensory inputs and projecting 10 trillion synapses onto 15 million PCs through PFs [14]. The GrC layer implements pattern separation through massive dimensional expansion, further enhanced by sparse coding mediated by local feedback inhibitions from Golgi cells (GoCs), in which only approximately 1%-10% of GrCs respond to any given sensory stimulus [15]. In addition, recent connectomic evidence has challenged the long-standing view that each GrC randomly integrates inputs from approximately 4 MFs. Instead, the structured organization in MF-GrC projections is characterized by non-random input-sharing motifs and over-representation patterns, where increased redundancy enhances resilience against noise [16].

A strikingly similar architectural and functional organization has been observed in the *Drosophila* mushroom body for odor coding, where the massive expansion in Kenyon cells (KCs) is regulated with feedback inhibition mediated by the anterior paired lateral (APL) neuron [17]. This evolutionary conservation suggests a computational principle where the winner-take-all (WTA) mechanism leads to the sparse encoding of sampled features, generating high-dimensional representations that maximize input orthogonality while preserving local structures [18]. Nonetheless, unlike the global WTA mediated by a single APL neuron in the *Drosophila* mushroom body [19,20], GoC feedback inhibition is a considerably spatially localized mechanism in the cerebellar circuitry, with each GoC inhibiting approximately 2000 GrCs and an estimated GoC-to-GrC ratio of 1:430 [21,22]. Furthermore, accumulating evidence indicates that the granular layer exhibits dynamic unsupervised learning mediated by multiple mechanisms, including the anti-Hebbian plasticity in GoC-mediated feedback inhibition [23–25], which has been proposed to enhance the sparse coding by promoting sparse representations and patterns decorrelation [26,27]. Collectively, these findings raise important questions regarding how to effectively incorporate the sparse coding scheme in a connectome-informed cerebellar model, and to what extent downstream performance might benefit from properties conferred by such sparse representations.

Here, we propose a cerebellum-inspired neural network model with three layers. The input layer performs resampling operations in which each GrC receives only a subset of input features. The granular layer generates expanded sparse representations through nonlinear WTA operations, utilizing tensor-based random MF-GrC weights combined with activity-dependent bias adjustments. This design resembles a standard single-hidden-layer feedforward neural network (FNN) architecture, aligning with established machine learning works [28] while incorporating key cerebellar anatomical and physiological features, including MF-GrC input-sharing connectivity and GoC-mediated localized feedback inhibition. Furthermore, by leveraging recent experimental discovery of intrinsic long-term potentiation (LTP) plasticity in PCs [8,24], integrated with several long-standing yet underappreciated observations, including the abundant interneuron population in CN and the baseline firing of CFs [29], the output layer is proposed with minimal hand-crafted designs to employ a robust supervised learning scheme that accommodates CF error signals to multi-class classification tasks. The proposed cerebellum-inspired neural network demonstrates comparable performance across various visual and motor tasks while achieving significant computational acceleration, compared to a conventional single-hidden-layer FNN trained via backpropagation.

## 2 Methods

### 2.1 Input preprocessing and mossy fiber sampling

In the visual tasks of Fashion-MNIST (fMNIST) and CIFAR-10, the input was normalized by dividing each pixel value by 255, the maximum grayscale value. For motor tasks, we applied Min-Max normalization to scale each feature to [0, 1]. Note that unless otherwise specified, all following paragraphs use row vectors.

To model the sparse input-sharing connectivity between MFs and GrCs, we employed an MF sampling scheme. Specifically, a preprocessed *d*-dimensional input vector 𝐱 is resampled *n* times with *m* features sampled in each iteration, reshaping $𝐱\in {\mathbb{R}}^{d}$ into a matrix $𝐗\in {\mathbb{R}}^{n\times m}$. For image datasets of fMNIST and CIFAR-10, we adopted a combined sampling strategy. In addition to random selection of *m* = 4 unique pixels, we incorporated local receptive-field (RF) sampling using 2×2 sliding windows. In visual tasks, this combined strategy exhaustively applies to all possible sliding windows for local RF sampling, followed by random sampling to complete the remaining iterations. For non-visual tasks, we implemented a generalized sampling strategy, where each RF is the unique combination of *m* components from the input vector 𝐱. Note that the connectivity topology between MFs and GrCs is determined by this sampling process with random initialization and remains fixed thereafter.

### 2.2 Tensor-based sparse coding in the granular layer

**Fig 2:**
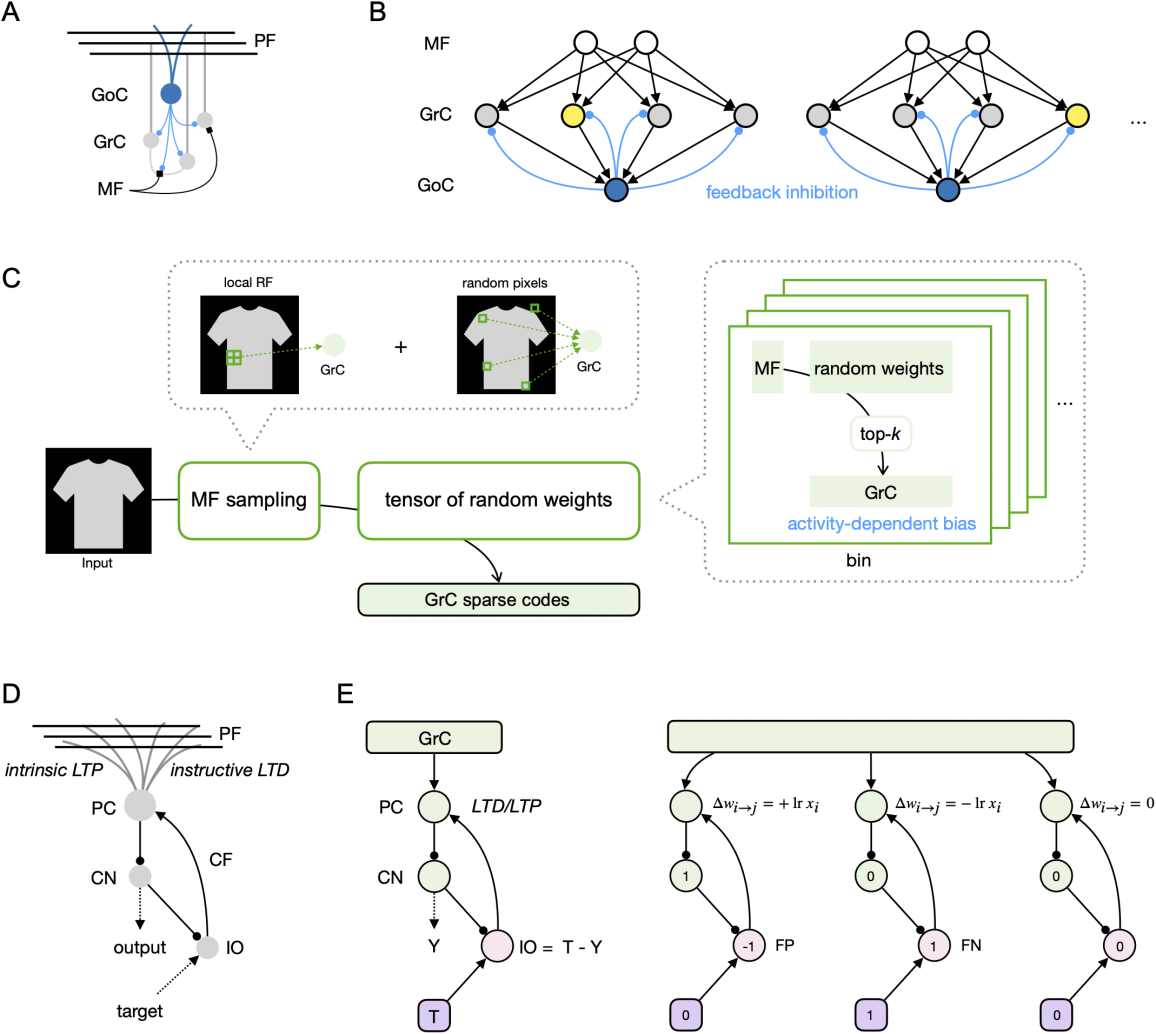
Scheme of sparse coding in the granular layer and learning principles in the Purkinje layer. **(A)** The upstream circuitry in the cerebellum, including excitatory MF inputs and inhibitory feedback inhibition mediated by GoCs to GrCs. **(B)** Sparse coding diagram of the upstream circuitry, illustrated with 2 independent bins. Yellow nodes indicate the winners in GrCs. **(C)** Schematic implementation of the tensor-based GrC sparse coding on visual tasks. **(D)** The downstream circuitry in the cerebellum, including PFs, PCs, CNs, IO, and CFs. Note the inhibitory nature of PC-CN and CN-IO synapses. **(E)** CF-error-driven supervised learning by integrated instructive LTD and intrinsic LTP, illustrating the distinction between FP and FN errors.

Utilizing localized feedback inhibitions mediated by GoCs, we developed a tensor-based sparse coding scheme defined as follows (Fig 2A, 2B and 2C). A *bin* is defined as a spatially localized group of GrCs that share identical sampled MF inputs and implement localized WTA through a specifically associated GoC. Consider a system with *n* bins, each containing *l* GrCs, yielding a total population of N=n×l GrCs. The number of MF sampling iterations equals the number of bins, with *m*-dimensional MF input shared within each bin, producing an input matrix $𝐗\in {\mathbb{R}}^{n\times m}$. Let $𝐓\in {\mathbb{R}}^{n\times m\times l}$ denote random weights from MFs to GrCs across all bins, where each entry is independently and uniformly sampled from [0,1), followed by each weight vector of GrC normalized by its Euclidean norm, i.e., ${𝐓}_{i,\;\cdot \;,\;j}\;/\;\|{𝐓}_{i,\;\cdot \;,\;j}{\|}_{2}\;,\;i=1,...,n,\;j=1,...,l$. This commonly used weight normalization technique [30] is applied to ensure that GrC activations characterize MF input patterns rather than differences in the Euclidean norm of weight vectors. Let $𝐁\in {\mathbb{R}}^{n\times l}$ denote the zero-initialized matrix for bias terms of the *N* GrCs, and $𝐛\in {\mathbb{R}}^{N}$ correspondingly denote the flattened vector. For any two vectors $\xi^{\left(1\right)},\xi^{\left(2\right)}\in {\mathbb{R}}^{m}$, we denote their canonical inner product by $\langle \xi^{\left(1\right)},\xi^{\left(2\right)}\rangle$. The tensor-based integration of the input matrix 𝐗 generates a matrix $𝐒\in {\mathbb{R}}^{n\times l}$:


$$\begin{array}{c} {𝐒}_{i,j}=\;\langle {𝐗}_{i,\;\cdot }\;,\;{𝐓}_{i,\;\cdot \;,j}\rangle \;+\;{𝐁}_{i,j}\;,\;i=1,...,n,\;j=1,...,l\;. \end{array}$$ (1)

As described in the Introduction, GoCs receive input from GrCs and provide feedback inhibition, thereby silencing the less-activated GrCs and preserving the most activated ones, referred to as the WTA mechanism. In our model, this GoC-mediated WTA process is approximated by the top-k function for winner selection in each bin, where *k* denotes the number of winners, producing the sparse matrix ${𝐒}^{\prime }\in {\mathbb{R}}^{n\times l}$:


$$\begin{array}{c} \begin{array}{cc} {𝐒}_{i,j}^{\prime } & ={\left(\text{top-}k\left({𝐒}_{i,\;\cdot }\right)\right)}_{j}\\ & :=\left\{ \begin{array}{cc} 1, & \text{if }{𝐒}_{i,j}\text{ is among the }k\text{ largest entries of }{𝐒}_{i,\;\cdot }\\ 0, & \text{otherwise} \end{array}\right.\\ & \;,i=1,...,n,\;j=1,...,l\;. \end{array} \end{array}$$ (2)

Finally, the matrix ${𝐒}^{\prime }$ is flattened into a binary vector $𝐠\in {\left\{0,1\right\}}^{N}$, referred to as the *GrC code*. We will elaborate on the coding process with a geometric interpretation in the Results section. Additionally, we developed coordinate-based storage and multiplication algorithms for efficient GrC code processing, as detailed in the S1 Appendix.

### 2.3 Encoding vector modules in sparse codes

The scaling invariance of the top-*k* operation follows directly from:


$$\begin{array}{c} \text{top-}k\;\left(𝐱\right)\equiv \text{top-}k\;\left(\lambda 𝐱\right)\;,\;\forall \lambda \in {\mathbb{R}}^{+}\;. \end{array}$$ (3)

Applications in motor control, where the input vector modules information is crucial, require minor modifications of the GrC sparse coding framework. An additional constantly-firing MF is introduced in each bin of GrCs, which is characterized by a fixed input value of 1 and the constant connection weights of 1 to all GrCs within the bin, as a simulation of background input activity. The remaining MFs retain the input values of features and random weights to GrCs, followed by the tensor-based sparse coding process as previously described. In detail, each MF vector $\xi \in {\mathbb{R}}^{m}$ is augmented to $\left[\xi ,1\right]\in {\mathbb{R}}^{m+1}$, where the appended 1 represents a constantly-firing MF. Correspondingly, the weight matrix ${𝐓}_{i,\;\cdot \;,\;\cdot \;}\in {\mathbb{R}}^{m\times l}\;,\;i=1,...,n$ is extended to $\left[\begin{array}{c} {𝐓}_{i,\;\cdot \;,\;\cdot \;}\\ 1 \end{array}\right]\in {\mathbb{R}}^{\left(m+1\right)\times l}\;,\;i=1,...,n$ before normalization, with $1\in {\mathbb{R}}^{l}$ denoting constant weights of 1 to all GrCs in this bin.

### 2.4 Activity-dependent bias adjustment

Feedback inhibitory synapses from GoCs to GrCs have been proposed to serve a “fire more, inhibit more” regulatory function that converges the firing rates toward a common value for all GrCs. This effect, which facilitates firing patterns decorrelation and mitigates the dead-neuron problem inherent in sparse coding schemes, can be modeled as an unsupervised adjustment on the bias term of each GrC, referred to as the activity-dependent bias in the literature [28].

In this study, we implemented the unsupervised activity-dependent bias adjustment in batch processing. For a batch of *p* samples with corresponding GrC codes, the firing rates of all *N* GrCs are represented in vector $𝐟\in {\mathbb{R}}^{N}$:


$$\begin{array}{c} {𝐟}_{j}=\frac{1}{p}\sum_{i=1}^{p}{𝐠}_{j}^{\left(i\right)}\;,\;j=1,...,N\;, \end{array}$$ (4)

where ${𝐠}^{\left(i\right)}$ denotes the GrC code of the *i*-th sample. The GrCs bias term vector 𝐛 is updated by:


$$\begin{array}{c} {𝐛}_{j}\leftarrow {𝐛}_{j}+\eta \;\left(\frac{k}{l}-{𝐟}_{j}\right)\;,\;j=1,...,N\;, \end{array}$$ (5)

where η is the learning rate. $\frac{k}{l}$ represents the expected firing rate of each GrC, given *k* winners in *l* GrCs during WTA, as elaborated in the S1 Appendix. Note that the batched calculation of firing rate 𝐟 should be interpreted as a computational readout of rate-coded population activity in the granular layer, rather than a realistic process performed by biological synapses.

### 2.5 Purkinje-layer linear multi-class classification

We developed a linear multi-class classifier for supervised learning of GrC codes, incorporating cerebellum-inspired constraints and plasticity-motivated update rules as described below (Fig 2D). Note that for simplicity in description, the term CN refers to a functional aggregation of neurons for output, which may represent only a subset of the complete anatomical cerebellar nuclei structure. Consider there are *q* distinct classes, each corresponding to a processing stream comprising a PC and a CN. The GrC code 𝐠 is first integrated in the PCs before being transmitted to the CNs for final output. The dense parallel fiber connections from GrCs to PCs are represented by a weight matrix $𝐖\in {\mathbb{R}}^{N\times q}$, initialized with all entries set to 1. The linear integration $𝐳\in {\mathbb{R}}^{q}$ in the Purkinje layer is subsequently computed as:


$$\begin{array}{c} {𝐳}_{j}=\langle 𝐠,{𝐖}_{\cdot \;,j}\rangle \;,\;j=1,...,q\;. \end{array}$$ (6)

Given the extensive interneuron connectivity, the final output of CNs is modeled with the one-versus-all operation top-1 that generates the one-hot encoded vector $\hat{𝐲}\in {\mathbb{R}}^{q}$ for the predicted integer label $\hat{y}\in \{1,...,q\}$:


$$\begin{array}{c} \begin{array}{c} \hat{y}={\text{argmax}}_{j}\left(-{𝐳}_{j}\right)\;,\;j=1,...,q\;, \end{array} \end{array}$$ (7)

$$\begin{array}{c} \begin{array}{cc} {\hat{𝐲}}_{j} & ={\left(\text{top-}1\left(-𝐳\right)\right)}_{j}\\ & :=\left\{ \begin{array}{cc} 1, & \text{if }j=\hat{y}\\ 0, & \text{otherwise} \end{array}\right.\;,\;j=1,...,q\;, \end{array} \end{array}$$ (8)

where the negative sign represents the inhibitory nature of PCs.

### 2.6 Climbing-fiber-driven supervised learning

The output $\hat{𝐲}$ of CNs is transmitted to the IO through inhibitory projection neurons, interestingly introducing an inversion of sign. Subsequently, $\hat{𝐲}$ is used to compare with the ground-truth one-hot encoded label vector $y\in {\mathbb{R}}^{q}$ and compute the error signal $𝐲-\hat{𝐲}$:


$$\begin{array}{c} \begin{array}{c} {\left(𝐲-\hat{𝐲}\right)}_{j}=\left\{ \begin{array}{cc} -1, & \text{if}\;{𝐲}_{j}=0\;\text{and}\;{\hat{𝐲}}_{j}=1\\ 0, & \text{if}\;{𝐲}_{j}={\hat{𝐲}}_{j}\\ 1, & \text{if}\;{𝐲}_{j}=1\;\text{and}\;{\hat{𝐲}}_{j}=0 \end{array}\right.\;\;,\;j=1,...,q\;. \end{array} \end{array}$$ (9)

In the scenario of incorrect prediction, the term FN position refers to where ${𝐲}_{j}=1\;\text{and}\;{\hat{𝐲}}_{j}=0$, while the term FP position refers to where ${𝐲}_{j}=0\;\text{and}\;{\hat{𝐲}}_{j}=1$.

We first adopted the supervised learning process under the classic assumption using CF-error-driven LTD, as detailed in the S1 Appendix. To enable distinction of both FN and FP errors, an extended mechanism, which decreases weights to the FN position and increases weights to the FP position, is proposed with integrated instructive LTD and intrinsic LTP in a rate-based neural framework given the baseline firing rate of CFs, as detailed below (Fig 2E).

Let $1\in {\mathbb{R}}^{q}$ denote the all-ones vector of dimension *q*. Consider the CF baseline firing rate is 1 Hz and compute the error signals $e\in {\mathbb{R}}^{q}$ relative to this baseline:


$$\begin{array}{c} 𝐞=1+𝐲-\hat{𝐲}\;. \end{array}$$ (10)

The instructive LTD is modeled as decreases in weights when a pre-synaptic GrC activation coincides with a CF error signal:


$$\begin{array}{c} 𝐖\leftarrow 𝐖-\alpha \;{𝐠}^{⊤}𝐞\;, \end{array}$$ (11)

where α is the learning rate of LTD. We further define the intrinsic LTP as constant increases in weights by pre-synaptic GrC firing activities, independent of error signals:


$$\begin{array}{c} 𝐖\leftarrow 𝐖+\beta \;{𝐠}^{⊤}1\;, \end{array}$$ (12)

where β is the learning rate of LTP. Combining two plasticity mechanisms yields the composite learning rule:


$$\begin{array}{c} \begin{array}{cc} 𝐖\leftarrow \; & 𝐖-\alpha \;{𝐠}^{⊤}𝐞+\beta \;{𝐠}^{⊤}1\\ & =𝐖-\alpha \;{𝐠}^{⊤}\left(1+𝐲-\hat{𝐲}\right)+\beta \;{𝐠}^{⊤}1\;. \end{array} \end{array}$$ (13)

Under the assumption of balanced plasticity where α=β, the learning rule simplifies to:


$$\begin{array}{c} 𝐖\leftarrow 𝐖+\alpha \;{𝐠}^{⊤}\left(\hat{𝐲}-𝐲\right)\;. \end{array}$$ (14)

Let $y,\;\hat{y}\in \{1,...,q\}$ denote the true and predicted integer labels, respectively. Interestingly, our learning rule is equivalent to the Multi-Class Perceptron algorithm [31], which becomes evident when rewritten in the following update form:


$$\begin{array}{c} \begin{array}{cc} & \text{if}\;\hat{y}\text{≠}y:\\ & {𝐖}_{\cdot \;,\;\hat{y}}\leftarrow {𝐖}_{\cdot \;,\;\hat{y}}+\alpha \;{𝐠}^{⊤}\;,\\ & {𝐖}_{\cdot \;,\;y}\leftarrow {𝐖}_{\cdot \;,\;y}-\alpha \;{𝐠}^{⊤}\;. \end{array} \end{array}$$ (15)

Interestingly, it could be proven that, given an arbitrary non-zero LTD learning rate α≠0, the learning process is independent of the specific value of the LTP learning rate β≠0 (**Theorem 1** in the S1 Appendix). The learning process of our model is governed by the critical updating component ${𝐠}^{⊤}(𝐲-\hat{𝐲})$, whose preservation is necessary and sufficient for the weights update in Eq. (13). Therefore, setting α=β only constitutes a concise and intuitive formulation, rather than implying a biologically established balance between LTD and LTP learning rates. This theoretical property was verified numerically on the fMNIST task (S1 Fig).

### 2.7 Fully-connected feedforward neural network

We employed a three-layer, fully connected FNN with a single hidden layer as the control model. The term “multi-layer perceptron” (MLP) is deliberately avoided, as “perceptron” specifically denotes the Multi-Class Perceptron algorithm in our study. The number of hidden units ranged from 500 to 50,000 to incrementally increase the model parameters, in which training was conducted via backpropagation using the Adam optimizer. Note that the FNN was used as a complexity-matched baseline rather than as a state-of-the-art visual classifier, while comparison with deep neural networks is outside the scope of our study for cerebellum-inspired lightweight architecture.

### 2.8 Datasets and tasks

In our study, all models were implemented and trained using the Flax and JAX libraries in Python on an NVIDIA A100 GPU. To ensure numerical accuracy, unless otherwise specified, we applied 64-bit float/integer in all tasks except for the comparison tasks with execution time reported, where 32-bit float/integer was used to underline the requirement of computation speed in actual applications. Parallelized operators in JAX Just-in-Time (JIT) compilation were implemented for all models whenever feasible.

We first generated a 2-dimensional toy dataset for concept illustrations. We sampled points $\left(x,y\right)\in {\mathbb{R}}^{2}$ with coordinates drawn independently and uniformly from $\left[0,r_{2}\right)$, then retained points satisfying $r_{1}^{2}\leq x^{2}+y^{2}\leq r_{2}^{2}$. We labeled the set *P*_0_, corresponding to *r*_1_ = 0, *r*_2_ = 0.5, as class 0, and the set *P*_1_, corresponding to *r*_1_ = 1, *r*_2_ = 1.5, as class 1. Let card(P) denote the cardinality of set *P*. For each i∈{0,1}, we randomly selected the training subset $P_{i,\text{train}}\subset P_{i}$ such that $\text{card}\left(P_{i,\text{train}}\right)$ equals the nearest integer to $\frac{\text{card}\left(P_{i}\right)}{2}$, which was designed around 1000, with the remaining points assigned to the test set.

We subsequently evaluated the cerebellum-inspired neural network on visual classification tasks of two benchmark datasets. The first dataset is fMNIST, consisting of 60,000 training and 10,000 test grayscale images of size 28×28 across 10 clothing categories. The second benchmark dataset is CIFAR-10 with 50,000 training and 10,000 test RGB color images of shape 3×32×32 drawn from 10 object categories, providing a more challenging task than fMNIST while maintaining similar image dimensions and identical class cardinality.

We further investigated the performance of the cerebellum-inspired neural network on two motor control tasks. The first is balancing the CartPole system of classic control theory, simulated in the OpenAI Gymnasium environment [32]. The second is controlling the robotic arm to reach the target position in the Reacher, a more challenging system with potential applications, simulated within the Multi-Joint dynamics with Contact (MuJoCo) physics engine [33] with Gymnasium. The training and evaluation framework is presented below.

### 2.9 Training and evaluation on motor control tasks

#### Trial-and-error based training.

To investigate the learning capability of the cerebellum-inspired neural network on motor control tasks, we utilized a supervised-learning based procedure for model training. A supervised-learning dataset comprising observations and actions was first sampled from the environment using a heuristic, trial-and-error based approach. The heuristic approach used for dataset sampling was introduced as an abstraction of the cerebral trial-and-error process for action selection. Briefly, a local search was performed by exploring all possible choices of action at each timestep, as the optimal action is constrained to a finite set of states within the general classification framework. The locally optimal action was selected according to a predefined task-specific metric based on the observations obtained during the local search, with the resultant observation-action pair recorded as the training data. The cerebellar model was subsequently trained on these observation-action pairs. Note that this procedure is aimed at demonstrations of supervised learning from motor datasets, rather than biologically realistic motor learning through autonomous experience.

Specifically, for a given task, a discrete action states set *A* is selected from the possible action space, with an indexing mapping of each action state 𝐚∈A to a multi-class label. Based on the motor control objective, a heuristic strategy *S* is defined to assess optimal actions (e.g., minimizing the distance between the robotic arm and the target in the Reacher task). The agent subsequently employs a trial-and-error based search for a duration of timesteps within a trial. Starting from observation ${𝐱}^{\left(t\right)}$ given at time *t*, the agent explores all possible action state sequences for consecutive *r* steps $\left({𝐚}^{\left(t\right)},{𝐚}^{\left(t+1\right)},...,{𝐚}^{\left(t+r-1\right)}\right)$, where ${𝐚}^{\left(t\right)}$ deno*t*es the action at time *t*, to obtain observations ${𝐱}^{\left(t+r\right)}$, resetting to time *t* after each iteration of *r*-step sequence. With the heuristic s*t*rategy *S*, the optimal actions for these *r* steps (from *t* to t+r−1) are selected based on the observations: $\left\{{𝐱}^{\left(t\right)}\right\}\cup \left\{{𝐱}^{\left(t+r\right)}|{𝐚}^{\left(t\right)},{𝐚}^{\left(t+1\right)},...,{𝐚}^{\left(t+r-1\right)}\in A\right\}$.

Notably, it is inherently time-consuming to search all possible states, limiting this approach to initial data sampling for the necessary number of training data entries. Once well-trained on the sampled dataset, the cerebellar model would execute near-optimal actions far more rapidly, forming an abstraction of the cerebellar supervised learning of observation-action mapping based on the cerebral trial-and-error experiences.

#### Real-time evaluation.

We evaluated the trained model using real-time simulations in the dynamic environment built from physically realistic equations, aiming to provide a proof-of-principle demonstration for potential applications of our model as a lightweight, rapidly reactive module operating in a larger dynamic environment. Specifically, at each time *t*, the model receives an observation ${𝐱}_{t}$ from the environment, and generates an instantaneous output action ${𝐚}_{t}$ to the environment. The simulation subsequently advances by a single timestep (the numerical integration interval for physical equations), and the updated environment provides the subsequent observation ${𝐱}_{t+1}$ back to the model for determining the next action ${𝐚}_{t+1}$. Therefore, in the evaluation process, the model retains a dependence on previous actions through its continuous interaction with the environment. The detailed, task-dependent procedures for dataset sampling, training, and evaluation are presented in the Results section.

## 3 Results

### 3.1 Construction of the cerebellum-inspired neural network

To construct the cerebellum-inspired neural network, we first implemented minimal dataset preprocessing, employing only Min-Max normalization scaling features to the same range while avoiding negative values. Beyond conventional assumptions of random MF-GrC projections, recent connectome studies have identified structured connectivity patterns, including redundant input-sharing and over-representation motifs [16]. Specifically, each GrC receives approximately 4 MF inputs, with localized GrCs exhibiting preferential connections to shared MF sources. Additionally, the localized feedback inhibition provided by GoC suggests a localized form of WTA sparsification [15,22], similar to a recent study that applied top-1 function to grouped neurons [28]. Taken together, we conceptualized each localized group of GrCs as a bin, where all GrCs receive shared MF inputs and feedback inhibition from a dedicated GoC. Mathematically, a bin is represented by a randomly initialized and normalized weight matrix of dimension: input MFs (e.g., 4) × bin width (the number of GrCs in each bin), with the top-*k* operation applied over GrCs. Concatenating bins of sampled MF inputs yields the GrC sparse code in vector form.

To address the dead-neuron problem inherent in sparse coding schemes, we utilized an unsupervised learning process on GrC bias terms that suppress overactive GrCs and reactivate “dead” neurons, which is biologically consistent with the GoC-mediated feedback inhibition properties [24,25]. In our implementation, the sparsity follows the experimental observation from 1% to 10%, ensuring that each GrC activates in only a small fraction of samples after bias adjustments. Our approach effectively mitigates the dead-neuron problem while simultaneously promoting decorrelation among GrC firing patterns, as detailed in the S1 Appendix.

To facilitate multi-class classification in the downstream circuitry, we modeled the population coding in CNs through the top-1 operation to generate one-hot encoded outputs, aligning with single winner selection mediated with abundant interneurons. Additionally, an alternative biological mechanism for one-hot encoding could be achieved by introducing lateral inhibition between PCs [8].

Focusing on the assumption that CFs convey binary error signals, i.e., the presence or absence of complex spikes, we first modeled the supervised learning process in the Purkinje layer with a classic LTD-based weight update rule [34]. However, since LTD operates unidirectionally by decreasing synaptic weights, it could only correct FN errors. To address this limitation, we incorporated intrinsic plasticity at PF-PC synapses in a rate-based design, formally defined as the *Purkinje-Nuclei Classifier* in the S1 Appendix, which enables corrections of both the FN and FP errors. Additionally, we trained and evaluated an *LTD-only variant* detailed in the S1 Appendix, with the Purkinje-Nuclei Classifier displaying higher overall and per-class accuracy on the fMNIST task (S2 Fig).

Interestingly, the learning process of our model is equivalent to the Multi-Class Perceptron algorithm, which utilizes (stochastic) gradient descent for weights optimization to minimize the Multi-Class Perceptron Loss. Remarkably, the supervised learning process becomes independent of the learning rate α when initializing the weights with all entries set to the same constant (**Theorem 3** in the S1 Appendix). This property eliminates the need for tuning the crucial hyperparameter of learning rate, which is particularly advantageous for a simplified, lightweight neural network of supervised learning. For all subsequent tasks, our implementations use α=1, though any positive value would yield equivalent results due to the independence property.

### 3.2 Geometric interpretations for GrC sparse representations

We elaborated our modeling approach by theoretical insights and mathematical proofs, specifically investigating properties of GrC sparse representations.

Since sampling occurs independently per bin, we simplify the following discussion by focusing on a single bin, where $\xi \in {\left[0,1\right]}^{m}$ denotes the corresponding preprocessed MF vector. The vector ξ is mapped into $𝐬\in {\mathbb{R}}^{l}$ via random weights $𝐔\in {\mathbb{R}}^{m\times l}$ and subsequently binarized into sparse code $s^{\prime }\in \{0,1{\}}^{l}$ with top-k operation:


$$\begin{array}{c} \begin{array}{c} {𝐬}_{j}=\langle \xi \;,\;{𝐔}_{\;\cdot \;,j}\rangle \;\;,\;j=1,...,l\;, \end{array} \end{array}$$ (16)

$$\begin{array}{c} \begin{array}{cc} {𝐬}^{\prime } & =\text{top-}k\left(𝐬\right)\;. \end{array} \end{array}$$ (17)

Since each entry of 𝐔 is independently and uniformly sampled from [0,1) and subsequently normalized to column-wise unit module, 𝐔 effectively forms a dictionary composed of *l* random atoms in ${\left[0,1\right]}^{m}$. Crucially, the top-k operation exhibits scaling invariance, as the *k* largest entries remain unchanged for any scaled vector λ𝐬, where $\lambda \in {\mathbb{R}}^{+}$. Therefore, our coding scheme selects the *k* atoms with the highest cosine similarity values relative to the input and assigns them a fixed coefficient of 1.

This binary sparse coding scheme employs the cosine-similarity-based *k*-nearest neighbors (KNN) representation from a random dictionary. Let $\left\{\phi^{\left(1\right)},\phi^{\left(2\right)},...,\phi^{\left(k\right)}\right\}\subset 𝐔$ denote the *k*-nearest neighbors of ξ under cosine similarity. When the sparse coding dictionary 𝐔 is randomly selected with sufficiently large cardinality, it will almost certainly contain *k* symmetric atoms within a predefined proximity to any $\xi \in {\left[0,1\right]}^{m}$, which are selected as $\phi^{\left(1\right)},\phi^{\left(2\right)},...,\phi^{\left(k\right)}$. Consequently, within a proximity threshold, inputs $\xi^{\left(1\right)},\xi^{\left(2\right)}\in {\left[0,1\right]}^{m}$ produce GrC codes with significant overlap, while sufficiently distant inputs generate non-overlapping codes due to disjoint sets of nearest neighbors. This threshold is primarily governed by the sparsity $\frac{k}{l}$ and affected by sampling randomness and the specific positions of the inputs. Therefore, our coding scheme preserves local neighborhood structures while maximizing the average distance between codes for distinct inputs, except when the inputs are sufficiently close.

Interestingly, in neural networks, cosine similarity represents both the simplest and most feasible measure for the proposed encoding scheme. The scaling invariance inherent to both cosine similarity and the top-k operation aligns well with scaling-invariant contexts of natural scenes, especially visual task processing, where visual datasets exhibit inherent scaling invariance as multiplying the input image by a moderate scalar preserves its essential characteristics for classification. For motor control applications, where the observation vectors ξ and λξ represent fundamentally different system states, preserving vector module information becomes crucial. We proposed an approach utilizing cosine similarity calculated in ${\left[0,1\right]}^{m+1}$ to identify *k*-nearest neighbors of $\xi \in {\left[0,1\right]}^{m}$, as [ξ,1] and $\left[\lambda \;\xi ,1\right],\lambda \in {\mathbb{R}}^{+}$ possess different angular orientations for distinction. For illustration, when *m* = 2, the *k*-nearest neighbors form an elliptical region (S3A and S3B Fig).

**Fig 3:**
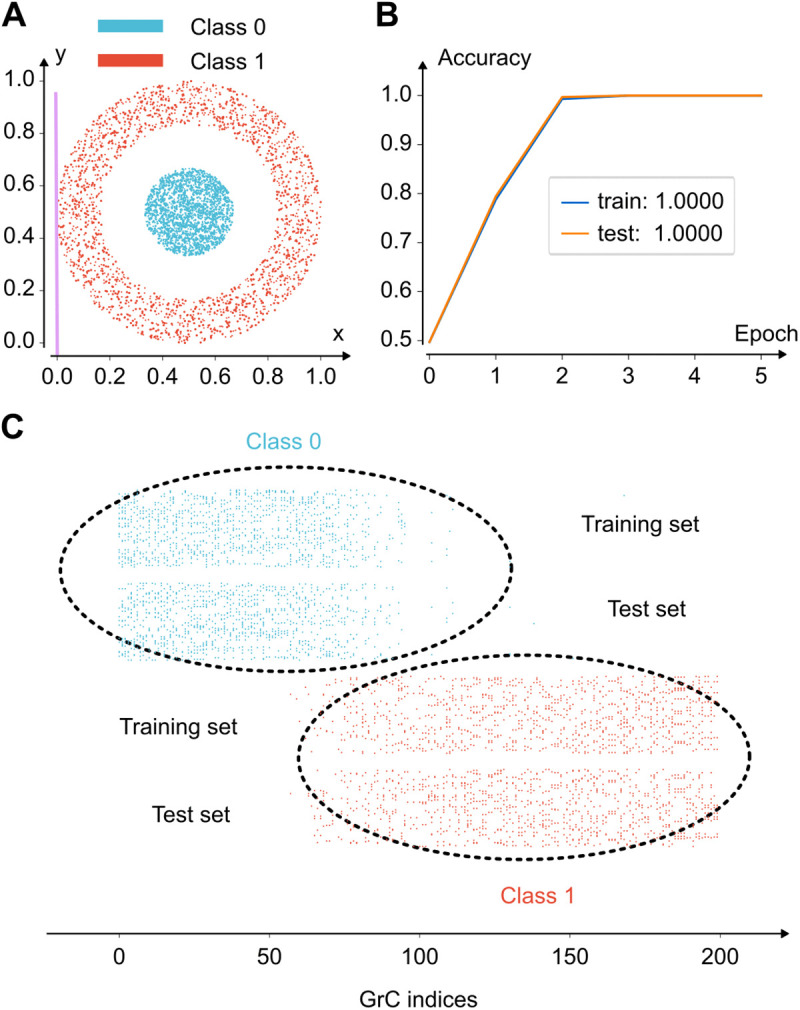
Expanded GrC sparse representation supports local-structure-preserving separation of input patterns. **(A)** Visualization of the 2-dimensional toy dataset after Min-Max normalization, with points in the test set twice as large. The purple line denotes the decision hyperplane of the Purkinje-Nuclei Classifier without GrC coding, with a training accuracy of approximately 50%. **(B)** The Purkinje-Nuclei Classifier achieved 100% accuracy on training and test sets after GrC coding. **(C)** The distinct firing pattern separation between two groups of GrCs, each representing a class, dramatically simplified the downstream classification.

We further demonstrated the GrC coding and Purkinje-layer learning principle using the following 2-dimensional toy dataset (Fig 3A). This scenario is linearly non-separable in ${\mathbb{R}}^{2}$, thus both the single-layer Multi-Class Perceptron and the Softmax Classifier (classic machine learning algorithm detailed in the S1 Appendix), despite incorporating bias terms, performed poorly (S4A Fig). However, the Euclidean-distance-based KNN Classifier achieved 100% test accuracy for various values of *k* (S4B Fig).

To demonstrate the efficacy of our method, we implemented a single bin configuration, with 10 winners from a total population of 200 GrCs. To eliminate dead GrCs, the activity-dependent bias adjustment was applied with learning rate 10^-3^ and batch size 100 over 20 epochs. The GrC codes were subsequently processed by the Purkinje-Nuclei Classifier for binary classification, achieving 100% accuracy on both training and test sets with batch size 10 after 5 epochs (Fig 3B). Therefore, our coding scheme successfully transformed the input patterns into the GrCs representation, which is separable by an origin-containing hyperplane. Furthermore, the GrC codes enabled 100% test accuracy when classified using the cosine-similarity-based KNN Classifier with various values of *k* (S4C Fig).

While it is well-established that projections to GrCs transform low-dimensional linearly non-separable inputs into high-dimensional separable representations, our analysis reveals a novel perspective that each GrC code represents an ensemble of *k*-cardinality sets of cosine-similarity-based *k*-nearest neighbors, rather than a point in the space with dimensionality equal to the total number of GrCs. Furthermore, by descendingly reordering GrCs according to their firing count differences between class 0 and class 1 training samples, we observed distinct firing pattern separation between two groups of GrCs, confirming that our GrC coding scheme preserves the local neighborhood structures and projects low-dimensional input patterns onto specific groups of components in the GrC coding vector (Fig 3C).

### 3.3 Performance on visual tasks

**Fig 4:**
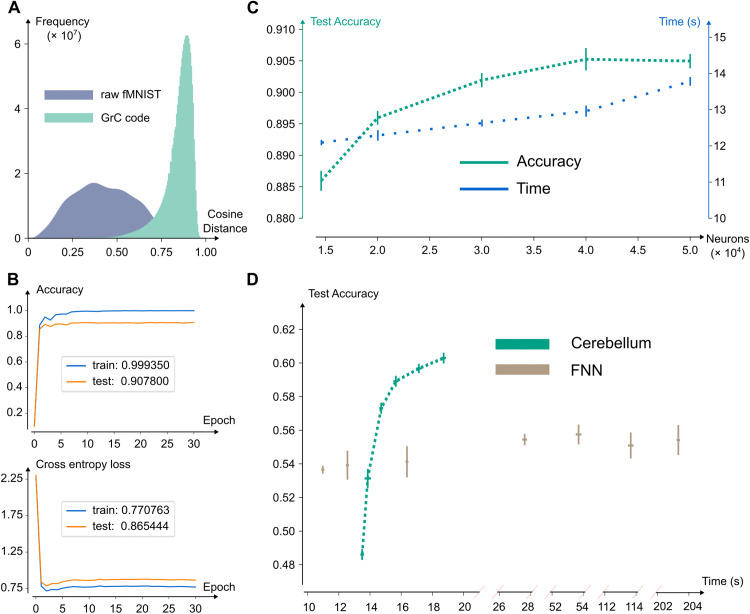
Performance of the cerebellum-inspired neural network on visual tasks. **(A)** The cosine distance distribution of inter-class paired input samples and GrC codes for the fMNIST training set. **(B)** The cerebellum-inspired neural network achieved a final test accuracy of 90.78% in a representative trial on the fMNIST task. Note that the cross-entropy loss was calculated with CN values that applied the Softmax function, while the top-1 function was applied in training. **(C)** The test accuracy and the script execution time of the cerebellum-inspired neural network on the fMNIST task. **(D)** The test accuracy associated with the script execution time of the cerebellum-inspired neural network and the FNN for a range of configurations on the CIFAR-10 task.

To evaluate the performance on the fMNIST dataset, we implemented the cerebellum-inspired neural network with 2500 bins, each containing 20 GrCs with 2 winners selected, yielding a total of 50,000 GrCs. The activity-dependent bias adjustment was applied with the learning rate 10^-3^ and batch size 100 for 5 epochs. The analysis of the cosine distance distribution of GrC codes displayed a larger average inter-class distance compared to the inputs (Fig 4A), while preserving the input local neighborhood structures (S5H and S5I Fig), similar to the patterns of previously illustrated 2-dimensional toy scenario. The Purkinje-Nuclei Classifier was subsequently trained with batch size 200 for 30 epochs, achieving a test accuracy of 90.78% (Fig 4B), which improved approximately 10% and 5% over the Multi-Class Perceptron and the Softmax Classifier, respectively, which were applied directly to fMNIST (S5A-D Fig). An ablation study was performed to elucidate the contributions of the receptive-field design, the bin architecture, and the activity-dependent bias adjustment to this performance gain (Table A in S1 Appendix). We further applied the Multi-Class Perceptron with bias, and the Softmax Classifier without and with bias on GrC codes, achieving minute improvements less than 0.5% (S5E, S5F, and S5G Fig), suggesting our approach is sufficiently effective in this task.

Additionally, a gap was observed between the training and the test accuracy, indicating imperfect generalization on the fMNIST task. This suggests that the sparse, high-dimensional GrC representation could effectively classify training samples, while its generalization on the test set requires further investigation. In the literature, the generalization performance depends on dataset complexity, especially the training-set coverage [35,36]. Following this interpretation, additional experiments on the fMNIST task, as detailed in the S1 Appendix, demonstrated that the test accuracy was significantly influenced by the test samples distribution compared with the training set (S6 Fig). The test accuracy also improved gradually as the training-set size increased (S7 Fig). Additionally, the generalization performance was slightly influenced by the downstream classifier. We analyzed a binary classification case to illustrate that the subtle performance gain of the Softmax Classifier might stem from its enhanced generalization capability by enlarging the decision margin (S8 Fig). These results indicate that the generalization performance of the present model on more complex datasets could presumably be improved through more adaptive sparse coding mechanisms or further architectural design of the classifier.

To emphasize the practical application of the cerebellum-inspired neural network, we exploited the properties of binary GrC codes and the learning rate independence theorem to develop efficient GrC code processing algorithms detailed in the S1 Appendix. Our implementation utilized 32-bit integer count matrices to eliminate numerical computation error while maintaining computational speed comparable to JAX-implemented matrix multiplication. We subsequently benchmarked the performance of our model on both fMNIST and CIFAR-10 datasets against a three-layer FNN trained with backpropagation using the Adam optimizer.

For the fMNIST task, we incrementally reduced the number of randomly sampled bins from 1771 to 0 while maintaining all 729 bins of local 2×2 RF, resulting in total bin configurations of 2500, 2000, 1500, 1000, 729 in our model, with identical training parameters as previously specified. We matched the hidden units of FNN to the number of GrCs, ranging from 50,000 to 14,580, and applied training with learning rate $2\times 10^{-4}$ and batch size 1000 for 100 epochs. For each sample in the CIFAR-10 dataset, we implemented GrC coding separately for each RGB channel and concatenated the 3 resulting GrC codes into a single vector with triple length for Purkinje-Nuclei Classifier processing. Each channel employed bin configurations of 3161, 2661, 2161, 1661, 1161, 961, with each bin containing 10 GrCs with 1 winner selected. The activity-dependent bias adjustment was applied with the learning rate 10^-3^ and batch size 100 for 5 epochs. Purkinje-Nuclei Classifier was subsequently trained with batch size 1000 for 40 epochs. The FNN was constructed with progressively decreasing hidden units: 50,000, 25,000, 10,000, 5000, 2000, 1000, 500, and was trained with learning rate $2\times 10^{-4}$ and batch size 1000 for 100 epochs. Note that for both models and tasks, these selected parameters were based on fine-tuning to achieve rapid training loss convergence. For each parameter configuration, numerical trials were conducted for 5 independent random seeds. During each trial, we recorded both the total Python script execution time and final test accuracy, reporting the mean ± standard deviation across 5 repetitions as our primary performance metric.

For both fMNIST and CIFAR-10 tasks, our model demonstrated comparable performance to the FNN and achieved computational acceleration with matching numbers of neurons (Figs 4C, 4D, and S9). Two factors primarily contribute to the rapid computation of our model. First, the GrC coding, equivalent to the input-to-hidden-layer transformation, operates as an unsupervised feedforward process. Therefore, our model requires only single Purkinje-layer matrix correction calculations, which are significantly faster than backpropagation through two layers. Second, by leveraging insights from the sparse codes and the gradient descent process, we developed efficient coordinate-based binary sparse GrC code storage and processing algorithms that enable parallel computation of samples, which contributes to the subtle time increase despite the number of GrCs scaled by more than 3 times. Therefore, our implementation maintained higher computational speed than FNN for larger numbers of neurons, achieving over 5× acceleration in the scenario with 50,000 GrCs or hidden units.

### 3.4 Performance on the CartPole task

**Fig 5:**
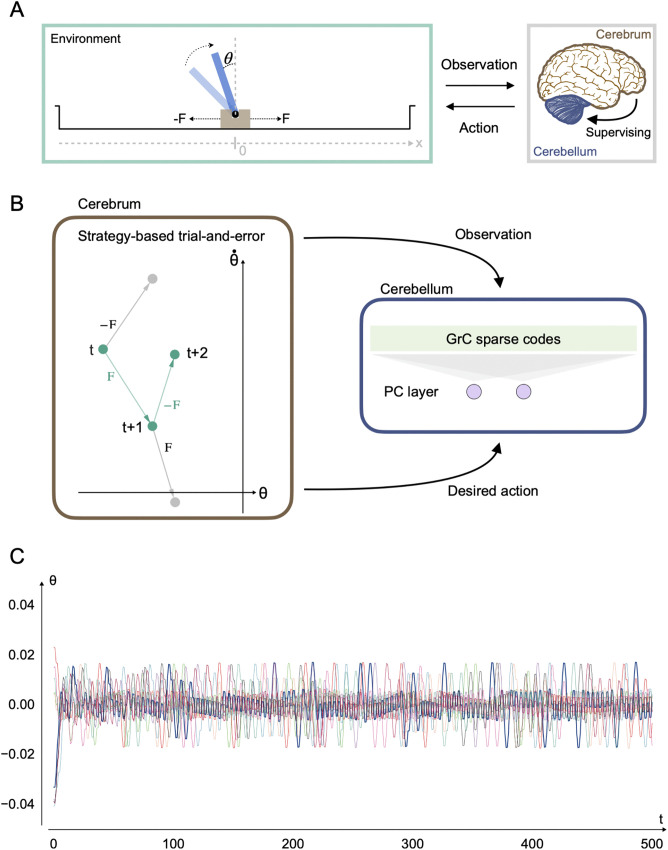
Cerebro-cerebellar framework for the cerebellum-inspired neural network on the CartPole task. **(A)** Illustrations of the CartPole environment and the cerebro-cerebellar framework. **(B)** Schematic diagram of the strategy-based trial-and-error for dataset generation and the training scheme. **(C)** The well-trained model rapidly stabilized the pole angular deviation from non-equilibrium initial states and maintained quasi-periodic subtle fluctuations for maximal achievable 500 timesteps. 10 randomly initialized trajectories were sampled for illustration.

We evaluated the performance of the cerebellum-inspired neural network on balancing the CartPole system with physically realistic dynamics simulated in the OpenAI Gymnasium environment (Fig 5A).

The CartPole environment consists of a cart moving horizontally along a frictionless track, with an unactuated pole attached via a frictionless hinge joint. The observation space incorporates four continuous variables: the cart’s linear displacement *x*^(*t*)^, the cart’s linear velocity ${\dot{x}}^{\left(t\right)}$, the pole’s angular displacement $\theta^{\left(t\right)}$, and the pole’s angular velocity ${\dot{\theta }}^{\left(t\right)}$. The discrete action space *A* contains binary force actuation: a constant leftward force ${𝐚}_{0}=-𝐅$ and a constant rightward force ${𝐚}_{1}=+𝐅$. The control objective is to select an action at each timestep to stabilize the system, with stability criteria imposing two constraints. First, the angular deviation of the pole must remain bounded ($|\theta^{\left(t\right)}|<12^{\circ }$). Second, the displacement of the cart must not exceed the track length (|*x*^(*t*)^| < 2.4). Trial termination occurs upon violation of either constraint. The performance metric is the duration of each trial, with maximal achievable 500 timesteps defining success.

We generated the dataset of observations and actions with the trial-and-error based strategy *S* defined as follows (Fig 5B). When selecting an action at time *t*, the agent applies ${𝐚}_{0}$ to obtain $\theta^{\left(t+1\right)}|\;{𝐚}_{0}$ and ${\dot{\theta }}^{\left(t+1\right)}|\;{𝐚}_{0}$, resets to the environment state at time *t*, then applies ${𝐚}_{1}$ to obtain $\theta^{\left(t+1\right)}|\;{𝐚}_{1}$ and ${\dot{\theta }}^{\left(t+1\right)}|\;{𝐚}_{1}$. For i∈{0,1}, *S* first selects ${\text{a}}_{i}$ if $\theta^{\left(t+1\right)}|\;{𝐚}_{i}$ and ${\dot{\theta }}^{\left(t+1\right)}|\;{𝐚}_{i}$ have different signs while $\theta^{\left(t+1\right)}|\;{𝐚}_{\left\{0,1\right\}⧵\left\{i\right\}}$ and ${\dot{\theta }}^{\left(t+1\right)}|\;{𝐚}_{\left\{0,1\right\}⧵\left\{i\right\}}$ have same signs. Otherwise, *S* selects ${𝐚}_{i}$ where $i={\text{argmin}}_{i}(|\left({\dot{\theta }}^{\left(t+1\right)}|\;{𝐚}_{i}\right)|)$. In the following text, all random initializations are employed by default methods of the Gymnasium environment. 1000 random seeds were sampled to initiate each trial, in which 200 timesteps of strategy-based trial-and-error were performed, with observation $\left(x^{\left(t\right)},{\dot{x}}^{\left(t\right)},\theta^{\left(t\right)},{\dot{\theta }}^{\left(t\right)}\right)$ and label of action (0 or 1) recorded at each timestep.

The generated dataset was partitioned into 190,000 training samples, with the remainder allocated to the validation set, followed by the GrC coding process. Each RF comprised 2 components from the 4-dimensional observation vector, generating $C_{4}^{2}=6$ bins, each populated by 1700 GrCs, resulting in a total of 10,200 GrCs. The top 5% of GrCs in each bin were selected as winners, and the activity-dependent bias adjustment was applied with learning rate 10^-4^ and batch size 100 for 10 epochs. Subsequently, the Purkinje-Nuclei Classifier was trained for binary classification using batch size 200 for 200 epochs.

Upon convergence of the training loss with a training accuracy of 100%, we evaluated the model performance in controlling the CartPole system within the Gymnasium environment across trials with random initializations. This evaluation of our model involved real-time continuous interaction directly with the environment, as detailed in the Methods section. The trained model successfully maintained balance for the maximal duration of 500 timesteps in all 100,000 trials without failure. Further analysis of dynamics revealed that the cerebellum-controlled pole rapidly stabilized from non-equilibrium initial states, exhibiting quasi-periodic fluctuations with subtle angular deviation (Fig 5C and S1 Video). Notably, the model was trained on a limited set of 1000 sampled trajectories but was systematically evaluated with randomly initialized, extensive 100,000 trials with sustained robust performance. Therefore, our model efficiently acquired and executed an observation-action mapping after supervised learning in the CartPole balancing task.

### 3.5 Performance on the Reacher task

**Fig 6:**
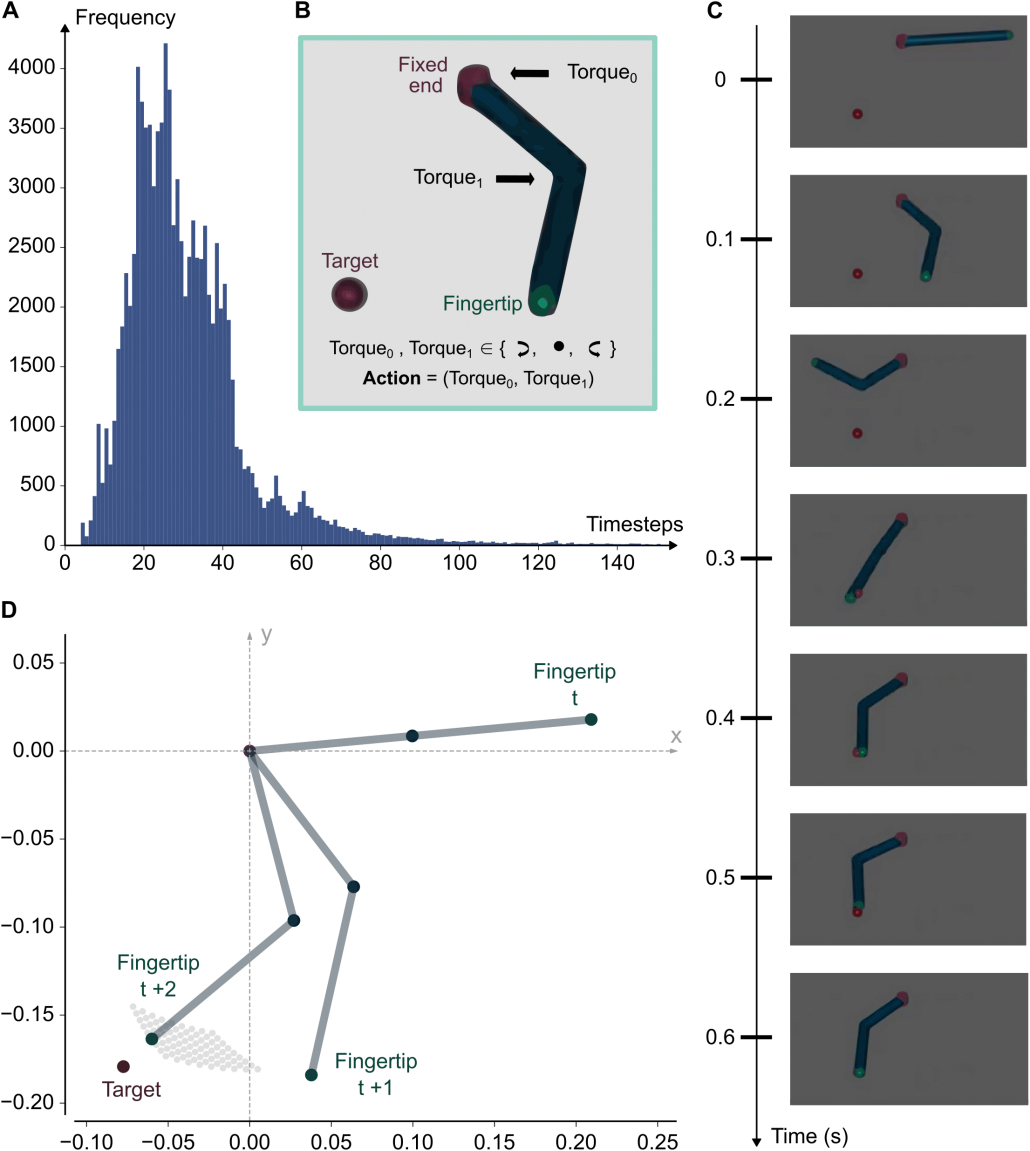
Performance of the cerebellum-inspired neural network on the Reacher task. **(A)** The trained model achieved a success rate of 99.14% across 100,000 randomly initialized trials, with a median termination time of 0.54 seconds for successful trials. **(B)** Illustration of the Reacher environment. **(C)** The snapshots of the well-trained model controlling the Reacher system. **(D)** Illustration of the trial-and-error based strategy for two consecutive steps in the Reacher.

We further evaluated the performance of the cerebellum-inspired neural network on the Reacher, a more challenging control system with realistic applications, simulated in the MuJoCo physics engine with Gymnasium (Fig 6B).

The Reacher environment involves controlling a two-joint robotic arm fixed at the origin (0, 0). The observation space consists of 10-dimensional continuous vector ${𝐱}^{\left(t\right)}$ with dependent components, while the action space comprises 2-dimensional continuous action vector $𝐚=(Torque_{0},Torque_{1})$ representing the torques applied at each hinge joint. The primary control objective is to minimize the time for the endpoint of the arm, referred to as the fingertip, to reach a randomly positioned target and subsequently stabilize it within a predefined proximity threshold. Note that both fingertip and target positions are randomly initialized at each trial onset, with the target remaining static thereafter.

We generated datasets of observations and actions with the trial-and-error based strategy *S* defined as follows (Fig 6D). Both *Torque*_0_ and *Torque*_1_ are constrained to discrete values chosen from {−1,0,1}. The discrete action space comprises all 9 possible combinations of these torques, defined as A={−1,0,1}×{−1,0,1}, with each action state labeled numerically from 0 to 8. Let *d*^(*t*)^ denote the Euclidean distance between the fingertip and the target at time *t*. When selecting actions at time *t* with observation ${𝐱}^{\left(t\right)}$, the agent explores all possible action state sequences for two consecutive steps to obtain $\left\{\left[d^{\left(t+2\right)}|\;{𝐱}^{\left(t\right)},\left({𝐚}^{\left(t\right)},{𝐚}^{\left(t+1\right)}\right)\right]|\;{𝐚}^{\left(t\right)},{𝐚}^{\left(t+1\right)}\in A\right\}$, resetting to time *t* after each execution of two-step action state sequence $\left({𝐚}^{\left(t\right)},{𝐚}^{\left(t+1\right)}\right)$. The strategy *S* is to select the $\left({𝐚}^{\left(t\right)},{𝐚}^{\left(t+1\right)}\right)$ which minimize $\left[d^{\left(t+2\right)}|\;{𝐱}^{\left(t\right)},\left({𝐚}^{\left(t\right)},{𝐚}^{\left(t+1\right)}\right)\right]$. We subsequently sampled 4000 random seeds to initiate each trial, in which 50 timesteps of trial-and-error were performed using *S*, with the observation ${𝐱}^{\left(t\right)}$ and corresponding action label (0–8) recorded at each timestep.

We implemented an analogous training procedure as described previously. The validation set comprised 100 samples, with the remaining samples allocated to the training dataset. For the GrC coding, each RF consisted of 5 components in the 10-dimensional observation vector, resulting in $C_{10}^{5}=252$ bins, each containing 400 GrCs with the top 5% selected as winners, yielding a total of 100,800 GrCs. The activity-dependent bias adjustment was applied with the learning rate 10^-4^ and batch size 100 for 10 epochs. The Purkinje-Nuclei Classifier was subsequently trained with batch size 200 for 800 epochs.

Upon convergence of the training loss, we evaluated the performance of the cerebellum-inspired neural network on the environment across 100,000 randomly initialized trials in real-time continuous interaction with the environment. Each trial spanned a maximum of 150 timesteps, with early termination triggered if *d*^(*t*)^ < 0.02 for 5 consecutive timesteps. Trials failing to satisfy this termination criterion within 150 timesteps were recorded as failures. For successful trials, the termination timestep served as the performance metric, which jointly assessed the speed of convergence to the target position and the steady-state positional maintenance stability. The trained model demonstrated robust performance, achieving a success rate of 99.14% with a median termination time of 27 timesteps for successful trials, which corresponds to 0.54 seconds in simulated environment time (Fig 6A and 6C and S2 Video). These results indicate that our model efficiently learned an observation-action mapping that supported closed-loop control of the Reacher system.

## 4 Discussion

Our study presents a comprehensive cerebellum-inspired neural network model incorporating unsupervised sparse coding and supervised learning mechanisms inspired by cerebellar circuits, achieving performance comparable to a single-hidden-layer backpropagation-trained FNN on benchmark visual tasks (e.g., > 90% test accuracy on fMNIST) [37,38]. Beyond comparable performance, our model demonstrates superior training efficiency to the FNN, primarily due to limiting the supervised learning to the downstream Purkinje layer and the fast implementation of our learning algorithm. These results demonstrate the potential of our cerebellum-inspired neural network as a lightweight and computationally efficient architecture promising for research direction, aiming at enhancing extensibility to more complex tasks while preserving the core advantage of rapid computation.

Furthermore, the proposed model exhibits robust learning efficiency in the proof-of-principle demonstration of supervised learning on motor tasks, in which the observation dataset was first sampled and subsequently used for training. Alternatively, we have proposed a real-time continuous learning approach, in which the model could be trained simultaneously with data collection using retrospective heuristic-based updates (S10 Fig). Following previous studies [39,40], our model could serve as a rapid-processing module of input-output mapping within a dynamic control system, advancing toward future applications of embedding lightweight cerebellum-inspired architectures into dynamically complex systems.

We achieve these advances by first organizing GrCs into localized groups with shared MF inputs, generating an ensemble of sparse representations on low-dimensional subsets of input features. In addition, we have employed the unsupervised activity-dependent bias adjustment, addressing the dead-neuron problem and promoting decorrelation of GrC activation patterns [41]. For generating sparse representations, the top-*k* operation is applied in each bin. Additionally, we have proposed an alternative computational implementation of WTA by overlapping GoC inhibitory fields in the granular layer (S11 Fig), illustrating that the non-overlapping top-*k* approach preserves robust feature representation capability while enabling substantially faster computation. The proposed model further enhances practical applicability with our efficient GrC code processing implementations that yield substantial computational acceleration. Additionally, our sparse binary coding and tensor-based design are well-suited for neuromorphic hardware implementation [42,43], offering particular potential in applications constrained by latency or limited computational resources.

Besides improving model performance, the bin design also extends the previous sparse coding study [18] in the cerebellum-inspired scheme while providing a clear geometric perspective. The scaling-invariant top-*k* operation simplifies the theoretical analysis compared with a previous study using rectified linear activation functions with shared thresholds across all GrCs [44]. In our design, each bin features a dense random-weights dictionary combined with WTA-based binary sparsification. Rather than viewing GrC sparse codes as isolated points in high-dimensional space, each code could be interpreted as *k*-cardinality sets of cosine-similarity-based *k*-nearest neighbors of the input in the low-dimensional space. Our perspective intuitively delineates properties of orthogonalizing most sample pairs unless they are sufficiently close, while also preserving local neighborhood structure, which is a well-established important property of sparse coding in previous studies.

In contrast to previous approaches incorporating Hebbian learning [28,45] or backpropagation-based training [38], our study demonstrates that untrained random weights in ensembled receptive-field architectures in the granular layer are sufficient to achieve significant coding performance across task domains ranging from visual to motor. This could be simply explained with our geometric interpretation, where sufficiently large number of random weight vectors form a sparse coding dictionary that is almost certain to cover the input space. Our model shares architectural similarities with the established lightweight framework of Extreme Learning Machine (ELM), which utilizes a fully connected single hidden layer fixed after random initialization, followed by a trainable linear classifier. Nevertheless, our top-*k* based bin design inherently generates sparse hidden-layer representations with a novel geometric interpretation and achieves improved performance on visual tasks [46,47]. Additionally, future research should elucidate the complex plasticity rules governing the granular layer, particularly investigating whether Hebbian-based adjustment of random-feature-based dictionary could enhance local structure preservation.

Interestingly, while conventional neural network theories rely on trainable input-to-hidden-layer weights [48,49], our study suggests that the cerebellum might employ an alternative strategy utilizing sufficiently wide granular layer to form a large random-weights dictionary [50], supported by the massive expansion of approximately 50 billion GrCs in humans, while addressing concerns regarding the biological plausibility of backpropagation in the brain [51]. In contrast to the “black-box” nature of traditional multi-layer neural networks trained via backpropagation, our model could be viewed as a “white-box” architecture, which implements random-feature-based sparse coding that could be interpreted using KNN-based geometric representations, followed by the Purkinje-Nuclei Classifier for supervised learning with mathematical properties and practical fast algorithms detailed in the S1 Appendix.

Additionally, the computational role of “bottleneck” architecture widely observed in cerebellum-like structures [52], including the *Drosophila* olfactory system, remains underexplored. Further introducing a compression layer between MF inputs and the GrC feature expansion layer might improve the efficiency of sparse coding and facilitate downstream learning.

By incorporating intrinsic plasticity in PF-PC synapses, we demonstrate that the Purkinje layer functions efficiently for multi-class classification, offering new insights into resolving the persistent challenge of learning inefficiency under binary instructive CF error signals. Our rate-based design utilizes mechanisms of baseline-dependent CF error signals incorporated with the intrinsic LTP, ensuring efficient weight adjustments in the Purkinje layer. This directly aligns with recent evidence from two-photon calcium imaging on PCs, which reveals that the complex spike rate encodes directions of sensorimotor errors, as the firing rate is elevated above baseline for a given error while suppressed below baseline when the error is inverted [53]. Previous studies also suggest that both reward delivery and omission signals are encoded by CFs [54,55]. Additionally, connections between KCs and mushroom-body output neurons in *Drosophila* undergo dopamine-mediated instructive LTD, and a recent study has also introduced LTP to encode both FN and FP prediction errors [56].

Beyond our simplified rate-based formulation for PCs, future study could explore a spiking variant of the Purkinje-Nuclei Classifier. For preliminary investigations, we have demonstrated that the computational principles from our static rate-based model, especially the Purkinje-Nuclei Classifier derived learning rule, could be extended to a dynamic spiking framework while maintaining comparable accuracy on the classification task (S12 Fig). Additionally, an alternative spiking model of supervised learning could presumably be implemented via inverse-Bienenstock-Cooper-Munro (iBCM) plasticity at PF-PC synapses, where simple spikes and complex spikes regulate the LTP/LTD switch through calcium dynamics [57]. Collectively, at both the rate and spiking levels, future study designs could further investigate learning schemes utilizing integrated instructive signals and intrinsic plasticity, as experimental evidence has revealed that parasagittal bands of PCs undergo different directions of plasticity in a synergistic learning process [58].

Interestingly, the perceptron hypothesis for PCs has long been proposed, dating back to the seminal work of Marr and Albus. Subsequent work by Brunel et al. showed that PC synaptic weight distributions align with analytical predictions for a sign-constrained perceptron at maximal capacity [59]. By integrating perspectives of error signals and synaptic plasticity, our study independently establishes a learning framework equivalent to the Multi-Class Perceptron algorithm, bridging theoretical and experimental advances in perceptron-like learning mechanisms for cerebellar models. As the Softmax Classifier slightly outperforms the Purkinje-Nuclei Classifier by enlarging the decision margin, future work could explore supervised learning mechanisms incorporating margin information in the Purkinje layer, extending further beyond the perceptron-like learning mechanisms. Lastly, assuming graded rate-based error signals, our modeling framework could be extended to a preliminary cerebellar model for regression (S13 Fig).

## 5 Limitations

Our study has several limitations. First, while the tensor-based implementation is computationally efficient, the grouping of GrCs into bins remains an oversimplification. In cerebellar circuits, GoC-mediated feedback inhibition exhibits greater complexity, including overlapping, spatially graded inhibitory fields on GrCs and lateral inhibitions among GoCs. Our method of directly selecting the *k* largest entries within each bin is a simplistic implementation of the GoC-mediated WTA process without finer-grained connectivity details, adopted to rapidly generate sparse GrC codes for downstream processing. Future work could explore more sophisticated GoC-GrC circuit architectures, presumably incorporating a dedicated GoC layer with connectome-informed inhibitory feedback. Second, the current model adopts a static, rate-based implementation to elucidate core computational principles while maintaining computational efficiency. Specifically, our modeling utilizes a feedforward architecture of sparse coding and supervised learning inspired by essential cerebellar computational principles, and does not provide a biologically realistic simulation of dynamics in cerebellar neural tissue. Future work should advance toward a biophysically realistic and dynamic spiking neural network with intricate nonlinear dendritic integration and Spike-Timing-Dependent Plasticity (STDP). Third, the learning mechanism in PCs and CNs, which is equivalent to the Multi-Class Perceptron algorithm, necessitates further experimental validation, especially given the intricate coding mechanisms observed in CN neurons [60]. Particularly, we utilize one-hot encoded label as an abstract supervised teaching signal compared with CN output in the IO, without addressing the biological sources of such signals. With diverse perspectives regarding the mechanisms of encoding and transmitting motor error signals [61–63], future studies should further investigate cortical circuits and olivary pathways related to motor control, aiming toward biologically realistic modeling for generating teaching signals. Finally, predefined strategies are employed to generate observation-action datasets for supervised learning in motor control tasks. While adequate for proof-of-principle validation, a more advanced cerebro-cerebellar framework would integrate recurrent neural networks with reinforcement online learning paradigm, serving as a real-time actor-critic design.

## 6 Conclusion

In conclusion, our study highlights cerebellum-inspired mechanisms of random-feature-based sparse representations in the granular layer, and CF-error-driven supervised learning for multi-class classification in the Purkinje layer. The proposed cerebellum-inspired neural network demonstrates robust performance across visual and simplified motor tasks, providing a novel perspective on how cerebellar computational principles may inspire lightweight neural network architectures.

## Supporting information

S1 Appendix Formal definitions, theoretical proofs, practical algorithms, and preliminary investigations of future research directions regarding the cerebellum-inspired neural network model. (PDF)

S1 FigThe performance of the Purkinje-Nuclei Classifier remained unaffected for a range of plasticity ratios $\frac{\beta }{\alpha }$.All configurations used identical hyperparameters except the plasticity ratio.(TIF)

S2 Fig Experiments regarding synaptic plasticity variants on the fMNIST task. (A)-(B) The per-class accuracy breakdown of the Purkinje-Nuclei Classifier (LTD + LTP) versus the LTD-only variant for training and test accuracy, respectively.
(TIF)

S3 FigCosine-similarity-based KNN representations in the GrC coding scheme.(A) Illustration of neighborhoods for two center points in ${\mathbb{R}}^{2}$. Elliptical regions represent the *k*-nearest neighbors of each center in red or blue. (B) Neighborhoods of sampled centers across the [0,1]^2^ region, with alternating red and blue coloring for clarity. (C) UMAP illustration of neighborhoods for sampled centers in ${\mathbb{R}}^{5}$. Note that Panels (B) and (C) correspond to the RFs in the CartPole and the Reacher tasks, respectively.(TIF)

S4 FigPerformance of various classifiers on the 2-dimensional toy dataset.(A) The decision boundaries are indicated in purple lines for three models: Multi-Class Perceptron with bias term (left), and Softmax Classifier without (middle) and with (right) bias term. All models exhibited poor accuracy around 50% due to the linear non-separability of the dataset in ${\mathbb{R}}^{2}$. (B) Directly applying the Euclidean-distance-based KNN Classifier resulted in 100% test accuracy across different *k* values. (C) The cosine-similarity-based KNN Classifier using GrC codes also attained 100% test accuracy for various *k* values.(TIF)

S5 Fig Performance of various classifiers on the fMNIST dataset. (A-D) Performance comparison of four baseline models applied directly to the fMNIST dataset: Multi-Class Perceptron without and with bias term, and Softmax Classifier without and with bias term. (E-G) Performances of three models applied to GrC codes: Multi-Class Perceptron with bias term, and Softmax Classifier without and with bias term. (H-I) Performance of the cosine-similarity-based KNN Classifier using raw fMNIST inputs or GrC codes, respectively.
(TIF)

S6 Fig The effect of sample distributions on the fMNIST task. (A)-(B) The UMAP visualization (colored in train/test) for the raw fMNIST whole dataset and the reshuffled fMNIST whole dataset, respectively. (C) Our model achieved a test accuracy significantly higher than the original after the reshuffling process.
(TIF)

S7 FigThe accuracy associated with the training-set size for a range of configurations on the fMNIST task.The mean ± standard deviation across 10 randomly initialized trials is reported for each training-set size.(TIF)

S8 Fig Investigation of performance gains in the Softmax classifier. We selected two classes, i.e., “sneaker” and “ankle boot”, from the fMNIST dataset for binary classification. Panel (A) displays the UMAP visualization of the corresponding fMNIST subsets. (B) Performance of our cerebellar model (left), comprising GrC coding followed by Purkinje-Nuclei Classifier training, with the distance of each sample to the decision boundary (right). In the histogram, training and test sets are shown in lighter and darker colors, respectively, for distinction. (C) The performance of the Softmax Classifier applied on GrC codes (left), with the distance of each sample to the decision boundary (right). Training and test sets maintain the same color scheme as above. Note that a subtle improvement (less than 0.5%) in test accuracy and a larger decision margin are observed in the Softmax Classifier.
(TIF)

S9 Fig Performance comparison between the cerebellar model and the FNN across different configurations on the fMNIST and the CIFAR-10 tasks. (A-D) Performance of four models on the fMNIST tasks: the cerebellar model with 14,580 GrCs, the FNN with 14,580 hidden units, the cerebellar model with 50,000 GrCs, and the FNN with 50,000 hidden units. (E-H) Performance of four models on the CIFAR-10 tasks: the cerebellar model with 28,830 GrCs, the FNN with 500 hidden units, the cerebellar model with 94,830 GrCs, and the FNN with 50,000 hidden units. (I-J) The test accuracy and the script execution time of the FNN on the fMNIST task.
(TIF)

S10 FigThe original and real-time continuous learning approaches for the CartPole task.(A) Schematic of the *n*-step simulation-and-training process in the real-time continuous learning approach, with each point representing a position of the cerebellum-controlled CartPole system in the phase space. (B) The evaluation duration associated with the training duration of different approaches. The mean ± standard deviation across 10,000 randomly initialized trials was evaluated after selected training durations.(TIF)

S11 Fig The functional similarity of the top-*k* implementation to overlapping GoC inhibition cases on the MNIST task. (A) Schematic implementation of the granular layer with overlapping GoC inhibitory fields, illustrated in the connection matrix. (B) The GrC firing distributions of the test set for different models. “Our” denotes the original top-*k* implementation that remains the primary focus of our study, and “GoC d = 0” denotes the overlapping inhibition case with the overlapping degree *d* = 0 (analogous for other *d* values). (C) Test accuracy of different models, displaying no statistically significant differences between the top-*k* implementation and the overlapping inhibition cases. (D) Python script execution time of different models, with the top-*k* implementation substantially faster.
(TIF)

S12 FigExtending rate-based model to cerebellum-inspired dynamic spiking framework for the Purkinje layer.(A) Schematic of the original rate-based model. (B) Schematic of the spiking framework, illustrating dynamics of the total synaptic conductance $g_{\text{syn}}\left(t\right)$ and the membrane potential v(t). Red and purple arrows indicate two EPSCs with different strengths and timings from two GrCs. (C) Training and test accuracy for the rate-based model (left) and the spiking model (right) on the binary classification case. (D) Total execution time of a 10,000-sample (batch size 500) forward simulation across model configurations. (E) Dynamic responses of two PCs to a single sample stimulus (from 0 to 50 ms), displaying post-stimulus differential conductance driving distinct output spike counts.(TIF)

S13 Fig Performance comparison between the Cerebellar Model for Regression (A) and the FNN (B) on the California Housing Dataset regression task. (TIF)

S1 Video Illustration of the model-controlled CartPole system in 10 randomly initialized trials. (MP4)

S2 Video Illustration of the model-controlled Reacher system in 10 randomly initialized trials. (MP4)
