# Supplementary material for: Cerebellum-inspired neural network of supervised learning with tensor-based sparse coding for multi-class classification
Source: PLoS Comput Biol. 2026 Aug 18;22(8):e1014595. doi: 10.1371/journal.pcbi.1014595 (PMC13485046; doi:10.1371/journal.pcbi.1014595)
Supplement: Supplementary file 1 [file pcbi.1014595.s001.pdf]

Cerebellum-inspired neural network of supervised learning with tensor-based sparse coding for multi-class classification

S1 Appendix

In the Supporting Information, we formally define the following classification models: the *Purkinje-Nuclei Classifier*, the *Multi-Class Perceptron with Bias*, the *Softmax Classifier*, and the *KNN Classifier*. The first three models are derived from the *Single-Layer Linear Classifier with Sign Inversion*.

We subsequently investigate their properties, as well as the properties of the *LTD-only variant of the Purkinje-Nuclei Classifier* and the *activity-dependent bias adjustment*, demonstrating theoretical insights of our cerebellum-inspired modeling presented in the main text. To emphasize the practical application of our model, we introduce *efficient GrC code processing algorithms*.

Additionally, we conduct preliminary investigations of future research directions based on the current model. First, aiming at enhancing performance on visual tasks, we investigate the *dataset-dependent generalization performance on visual tasks*. Second, we propose a *real-time continuous learning approach in motor tasks*, constituting an alternative to static training on sampled datasets as detailed in the main text. Third, given the intricate overlapping connectivity involved in the actual GoC-GrC circuit, we propose a *WTA implementation by overlapping GoC inhibitory fields* in the granular layer. Fourth, we demonstrate that the computational principles from our rate-based model could be extended to a *cerebellum-inspired dynamic spiking framework* while maintaining comparable accuracy on the classification task. Finally, we discuss the *Cerebellar Model for Regression*, provided CFs could transmit graded, rate-based signals.

Definition 1 (Single-Layer Linear Classifier)**.** *Consider P input samples of dimension N, and q distinct classes. Let $Y \in \mathbb{R}^P$ denote the ground-truth integer labels. The inputs and one-hot encoded labels are represented by matrices $\mathbf{X} \in \mathbb{R}^{P\times N}$ and $\mathbf{Y} \in \mathbb{R}^{P\times q}$, respectively, with each row corresponding to a single sample. Let $\mathbf{W}^{(r)} \in \mathbb{R}^{N\times q}$ denote the weight matrix from the input layer to the output layer, and $\mathbf{b}^{(r)} \in \mathbb{R}^q$ denote the bias term of the output layer <u>after</u> r epochs of training. <u>After</u> r epochs, the linear integration $\mathbf{Z}^{(r)} \in \mathbb{R}^{P\times q}$ is computed as:*

$$\mathbf{Z}^{(r)}_{i,\cdot} = (\mathbf{X}_{i,\cdot})\,\mathbf{W}^{(r)} + \mathbf{b}^{(r)} \ \ , \ \ i \in \{1,\dots,P\} \ \ . \tag{S1}$$

The final output is $\mathbf{Z}^{(r)}$. The predicted integer labels <u>after</u> r epochs are denoted as $\hat{\mathcal{Y}}^{(r)} \in \mathbb{R}^P$:

$$\hat{\mathcal{Y}}^{(r)}_i = \mathrm{argmax}_j(\mathbf{Z}^{(r)}_{i,j}) \ \ , \ \ i \in \{1,\dots,P\} \ \ . \tag{S2}$$

Definition 2 (Single-Layer Linear Classifier with Sign Inversion)**.** *Consider a Single-Layer Linear Classifier modified by introducing a sign inversion in the final output, i.e.,* $-\mathbf{Z}^{(r)}$*. The predicted integer labels and predicted one-hot encoded labels* after *r epochs are denoted as* $\hat{Y}^{(r)} \in \mathbb{R}^{P}$ *and* $\hat{\mathbf{Y}}^{(r)} \in \mathbb{R}^{P\times q}$*, respectively:*

$$\hat{Y}_i^{(r)} = \mathrm{argmax}_j(-\mathbf{Z}_{i,j}^{(r)}) \ \ , \ \ i \in \{1,\ldots,P\} \ \ , \tag{S3}$$

$$\hat{\mathbf{Y}}_{i,j}^{(r)} = \left(\text{top-1}\left(-\mathbf{Z}_{i,\cdot}^{(r)}\right)\right)_j \tag{S4}$$

$$:= \begin{cases} 1, & \text{if } j = \hat{Y}_i^{(r)} \\ 0, & \text{otherwise} \end{cases} \ \ , \ \ i \in \{1,\ldots,P\} \ \ , \ \ j \in \{1,\ldots,q\} \ \ . \tag{S5}$$

Definition 3 (Purkinje-Nuclei Classifier)**.** *Consider a Single-Layer Linear Classifier with Sign Inversion, in which the bias term is constant, i.e.,* $b \in \mathbb{R}\ ,\ \mathbf{b}_j^{(r)} \equiv b\ ,\ j \in \{1,\ldots,q\}$*. Notably, because of the translation invariance of the top-1 operation, any value of b would yield an equivalent result as $b = 0$. The Multi-Class Perceptron Loss* after *r epochs* $L^{(r)} \in \mathbb{R}$ *is defined as:*

$$L^{(r)} = \sum_{i=1}^{P}\left(\max_j\left(-\mathbf{Z}_{i,j}^{(r)}\right) + \mathbf{Z}_{i,Y_i}^{(r)}\right) \ \ . \tag{S6}$$

Let $\left(\frac{\partial L}{\partial \mathbf{W}}\right)^{(r)} \in \mathbb{R}^{N\times q}$ *denote the gradient of the loss function with respect to* $\mathbf{W}$ *calculated* at *the r-th epoch. Applying gradient descent optimization, the weight correction applied* at *the r-th epoch* $\Delta\mathbf{W}^{(r)} \in \mathbb{R}^{N\times q}$ *is computed as:*

$$\begin{aligned}\Delta\mathbf{W}^{(r)} &= -\alpha\left(\frac{\partial L}{\partial \mathbf{W}}\right)^{(r)} \\ &= \alpha\,\mathbf{X}^{\top}\left(\hat{\mathbf{Y}}^{(r-1)} - \mathbf{Y}\right) \ \ ,\end{aligned} \tag{S7}$$

where α is the learning rate. This can be rewritten in the following form of update algorithm:

$$\begin{aligned}&\text{for } i \in \{1,\ldots,P\} : \\ &\quad \text{if } \ \hat{Y}_i^{(r-1)} \neq Y_i : \\ &\qquad \mathbf{W}_{\cdot,\hat{Y}_i^{(r-1)}}^{(r)} = \mathbf{W}_{\cdot,\hat{Y}_i^{(r-1)}}^{(r-1)} + \alpha\left(\mathbf{X}_{i,\cdot}\right)^{\top} \ \ , \\ &\qquad \mathbf{W}_{\cdot,Y_i}^{(r)} = \mathbf{W}_{\cdot,Y_i}^{(r-1)} - \alpha\left(\mathbf{X}_{i,\cdot}\right)^{\top} \ \ .\end{aligned} \tag{S8}$$

Definition 4 (Multi-Class Perceptron with Bias). *The Multi-Class Perceptron with Bias is a model of Single-Layer Linear Classifier with Sign Inversion, in which both the weights and the bias terms are trainable parameters. Applying gradient descent optimization, the weight adjustment follows the established Eq.* (S7)*, and the bias adjustment applied at the r-th epoch $\Delta\mathbf{b}^{(r)} \in \mathbb{R}^q$ is calculated as:*

$$\Delta\mathbf{b}^{(r)} = \alpha \sum_{i=1}^{P} \left(\hat{\mathbf{Y}}_{i,\cdot}^{(r-1)} - \mathbf{Y}_{i,\cdot} \right) \quad . \tag{S9}$$

Theorem 1 (Independence of Long-Term Potentiation). *Let $\mathbf{1} \in \mathbb{R}^{P\times q}$ denote the all-ones matrix of dimension $P \times q$, and $\lambda \in \mathbb{R}$. For the Purkinje-Nuclei Classifier with an arbitrary learning rate $\alpha \neq 0$, if Eq.* (S7) *is modified to:*

$$\Delta\mathbf{W}^{(r)} = \alpha\, \mathbf{X}^\top \left(\hat{\mathbf{Y}}^{(r-1)} - \mathbf{Y} + \lambda\, \mathbf{1} \right) \quad , \tag{S10}$$

the weight adjustment established by Eq. (S10) *is independent of the coefficient λ.*

Proof. Let $\mathbf{U} \in \mathbb{R}^{N\times q}$ denote an arbitrary matrix with identical column vectors:

$$\mathbf{U} = \left[\mathbf{w}^\top, \mathbf{w}^\top, \ldots, \mathbf{w}^\top\right] \quad , \quad \forall \mathbf{w} \in \mathbb{R}^N \quad . \tag{S11}$$

Given the translation invariance of the top-1 operation:

$$\forall c \in \mathbb{R}, \;\; \forall \boldsymbol{\xi} \in \mathbb{R}^q, \;\; \text{top-1}\left(\boldsymbol{\xi} + c\right) \equiv \text{top-1}\left(\boldsymbol{\xi}\right) \quad , \tag{S12}$$

it is straightforward to verify that after arbitrary r epochs, replacing $\mathbf{W}^{(r)}$ with $\mathbf{W}^{(r)} + \mathbf{U}$ yields the same output $\hat{\mathbf{Y}}^{(r)}$. The discrepancy between Eq. (S7) and Eq. (S10) is the term $\alpha\, \mathbf{X}^\top \lambda\, \mathbf{1}$, which constitutes a specific instance of $\mathbf{U}$. By the principle of mathematical induction, the stated property holds for all integers $r \geq 0$. □

From **Theorem 1**, using $\lambda = \frac{\beta}{\alpha} - 1$, one can readily verify that the term $\beta\, \mathbf{g}^\top \mathbf{1}$ in Eq.(13) of the main text could be omitted without affecting the learning performance, thereby establishing the "Independence of Long-Term Potentiation".

Theorem 2 (Independence of Sign Inversion). *A Single-Layer Linear Classifier with Sign Inversion **Classifier**$_1$ generates identical output to a Single-Layer Linear Classifier **Classifier**$_2$, if **Classifier**$_1$ is initialized with sign-inverted weights and biases relative to **Classifier**$_2$ and trained with an identical learning rate.*

Proof. From the definition, one can readily verify that the theorem holds true by applying mathematical induction on the epochs. □

Theorem 3 (Independence of Learning Rate for Purkinje-Nuclei Classifier)**.** *If a Purkinje-Nuclei Classifier is initialized with:*

$$\mathbf{w} \in \mathbb{R}^N \ \ , \mathbf{W}^{(0)} = \left[\mathbf{w}^\top, \mathbf{w}^\top, \dots, \mathbf{w}^\top\right] \ \ , \tag{S13}$$

the (stochastic) gradient descent optimization is independent of the learning rate α.

Proof. We proceed by mathematical induction on the number of epochs r. We first have:

$$\forall i \in \{1, \dots, P\} \ , \forall j \in \{1, \dots, q\} \ , \mathbf{Z}_{i,j}^{(0)} = \langle \mathbf{X}_{i,\cdot} \ , \mathbf{w}^\top \rangle =: c_i \ \ . \tag{S14}$$

When assuming uniformly distributed samples across classes, without loss of generality, one may consider the Classifier assigning the first label to all samples. Therefore, the initial prediction $\hat{\mathbf{Y}}^{(0)}$ is independent of the learning rate α. Assume the statement holds for all integers $r \geq 0$. We shall prove it for the $(r+1)$-th epoch. For the training process, we have:

$$\mathbf{W}^{(r+1)} = \mathbf{W}^{(r)} + \alpha \mathbf{X}^\top \left(\hat{\mathbf{Y}}^{(r)} - \mathbf{Y}\right) \ \ . \tag{S15}$$

It is straightforward to verify that:

$$\mathbf{W}^{(r+1)} = \mathbf{W}^{(0)} + \alpha \mathbf{X}^\top \sum_{j=0}^{r} \left(\hat{\mathbf{Y}}^{(j)} - \mathbf{Y}\right) \ \ . \tag{S16}$$

For the output, we have:

$$\begin{aligned} \forall\, i \ \in \ \ & \{1, \dots, P\} \ , \\ & \mathbf{Z}_{i,\cdot}^{(r+1)} = \left(\mathbf{X}_{i,\cdot}\right) \mathbf{W}^{(r+1)} \\ & \quad = c_i + \ \alpha \ \left(\mathbf{X}_{i,\cdot}\right) \ \mathbf{X}^\top \sum_{j=0}^{r} \left(\hat{\mathbf{Y}}^{(j)} - \mathbf{Y}\right) \ \ . \end{aligned} \tag{S17}$$

To get predicted one-hot label, we have $\hat{\mathbf{Y}}_{i,\cdot}^{(r+1)} = \text{top-1}\left(-\mathbf{Z}_{i,\cdot}^{(r+1)}\right)$. Notably, as a special case of top-k, the top-1 operation has both the translation invariance and the scaling invariance:

$$\begin{aligned} & \forall\, \alpha\,, c \in \mathbb{R}\,, \alpha > 0, \ \ \forall\, \boldsymbol{\xi} \in \mathbb{R}^q, \\ & \text{top-1}\left(\alpha \boldsymbol{\xi} + c\right) \equiv \text{top-1}\left(\boldsymbol{\xi}\right) \ \ . \end{aligned} \tag{S18}$$

Therefore, we have:

$$\begin{aligned}
&\forall\, i \in \{1, \dots, P\} \;, \\
&\hat{\mathbf{Y}}^{(r+1)}_{i,\cdot} = \text{top-1}\left(-c_i - \alpha \left(\mathbf{X}_{i,\cdot}\right) \mathbf{X}^{\top} \sum_{j=0}^{r} \left(\hat{\mathbf{Y}}^{(j)} - \mathbf{Y}\right)\right) \\
&\qquad\;\; = \text{top-1}\left(\left(\mathbf{X}_{i,\cdot}\right) \mathbf{X}^{\top} \sum_{j=0}^{r} \left(\mathbf{Y} - \hat{\mathbf{Y}}^{(j)}\right)\right) \quad .
\end{aligned} \tag{S19}$$

By the assumption, $\sum_{j=0}^{r}\left(\mathbf{Y} - \hat{\mathbf{Y}}^{(j)}\right)$ is independent of α. Therefore, $\hat{\mathbf{Y}}^{(r+1)}$ is independent of α. By the principle of mathematical induction, the statement holds for all integers $r \geq 0$.

It is straightforward to verify that the similar proof holds true in the scenario of stochastic gradient descent. □

Theorem 4 (Independence of Learning Rate for Multi-Class Perceptron with Bias)**.** *If a Multi-Class Perceptron with Bias is initialized with:*

$$\begin{aligned}
&\mathbf{w} \in \mathbb{R}^{N} \;\;, \mathbf{W}^{(0)} = \left[\mathbf{w}^{\top}, \mathbf{w}^{\top}, \dots, \mathbf{w}^{\top}\right] \;\;, \\
&b \in \mathbb{R} \;\;, \mathbf{b}^{(0)} = [b, b, \dots, b] \;\;,
\end{aligned} \tag{S20}$$

the (stochastic) gradient descent optimization is independent of the learning rate α.

Proof. We use similar proof technique as **Theorem 3**. We first have:

$$\forall i \in \{1, \dots, P\} \;, \forall j \in \{1, \dots, q\} \;, \mathbf{Z}^{(0)}_{i,j} = \langle \mathbf{X}_{i,\cdot} \;, \mathbf{w}^{\top} \rangle + b =: c_i \;\; . \tag{S21}$$

Therefore, $\hat{\mathbf{Y}}^{(0)}$ is independent of α. Assume the statement holds for all integers $r \geq 0$. We shall prove it for the $(r+1)$-th epoch. One can readily verify that:

$$\begin{aligned}
&\forall\, i \in \{1, \dots, P\} \;, \\
&\mathbf{Z}^{(r+1)}_{i,\cdot} = c_i + \alpha \left(\mathbf{X}_{i,\cdot}\right) \mathbf{X}^{\top} \sum_{j=0}^{r} \left(\hat{\mathbf{Y}}^{(j)} - \mathbf{Y}\right) + \alpha \sum_{j=0}^{r} \sum_{k=1}^{P} \left(\hat{\mathbf{Y}}^{(j)}_{k,\cdot} - \mathbf{Y}_{k,\cdot}\right) \quad .
\end{aligned} \tag{S22}$$

Because of the translation invariance and the scaling invariance of the top-1 operation, $\hat{\mathbf{Y}}^{(r+1)}$ is independent of α. By the principle of mathematical induction, the statement holds for all integers $r \geq 0$. □

Theorem 5 (Converged Training Accuracy for Purkinje-Nuclei Classifier)**.** *Consider a Purkinje-Nuclei Classifier trained with gradient descent. Provided that the input matrix* $\mathbf{X}$ *possesses full row rank, the Classifier is certain to achieve* 100% *training accuracy upon convergence.*

Proof. From **Theorem 3** Eq. (S15), using $\alpha = 1$, we have:

$$\mathbf{W}^{(r+1)} = \mathbf{W}^{(r)} + \mathbf{X}^{\top}\left(\hat{\mathbf{Y}}^{(r)} - \mathbf{Y}\right) \quad . \tag{S23}$$

Provided that $\mathbf{X}$ has full row rank, we have $\hat{\mathbf{Y}}^{(+\infty)} = \mathbf{Y}$ upon convergence. □

Interestingly, the GrC codes serve as the input matrix to the Purkinje-Nuclei Classifier. We have demonstrated the GrC coding is a local-structure-preserving projection of input patterns onto specific groups of components in the GrC coding vector, which implies a high likelihood of the input matrix achieving full row rank with a sufficiently large number of GrCs. This may explain why the cerebellar model empirically exhibits training accuracy approaching 100% more readily than the FNN.

Definition 5 (Softmax Classifier)**.** *For each sample, the predicted one-hot encoded label* $\hat{\mathbf{Y}}^{(r)}_{i,\cdot}$ $, i \in \{1, \ldots, P\}$ *represents a "hard" probability distribution, assigning probability* 1 *to the predicted class and* 0 *to all others. In contrast, the Softmax Classifier generates a "soft" probabilistic assignment of classes by applying the Softmax function followed by optimization using the cross-entropy loss:*

$$\mathbf{U}^{(r)}_{i,j} = \left(\text{Softmax}\left(-\mathbf{Z}^{(r)}_{i,\cdot}\right)\right)_j \tag{S24}$$

$$:= \frac{e^{-\mathbf{Z}^{(r)}_{i,j}}}{\sum_{k=1}^{q}\left(e^{-\mathbf{Z}^{(r)}_{i,k}}\right)} \quad , \quad i \in \{1, \ldots, P\} \quad , \quad j \in \{1, \ldots, q\} \quad , \tag{S25}$$

$$L^{(r)}_{\text{cross-entropy}} = -\sum_{i=1}^{P}\sum_{j=1}^{q}\left(\mathbf{Y}_{i,j} \quad \cdot \quad \ln \mathbf{U}^{(r)}_{i,j}\right) \quad . \tag{S26}$$

The weights and bias gradient descent optimizations are calculated as:

$$\Delta\mathbf{W}^{(r)}_{\text{Softmax}} = \alpha\, \mathbf{X}^{\top}\left(\mathbf{U}^{(r-1)} - \mathbf{Y}\right) \quad , \tag{S27}$$

$$\Delta\mathbf{b}^{(r)}_{\text{Softmax}} = \alpha \sum_{i=1}^{P}\left(\mathbf{U}^{(r-1)}_{i,\cdot} - \mathbf{Y}_{i,\cdot}\right) \quad . \tag{S28}$$

Definition 6 (KNN Classifier)**.** *Let* D *denote a metric of distance in* $\mathbb{R}^N$*, and* $k \in \mathbb{Z}^+$*. For a test sample* $\mathbf{x}^{(0)} \in \mathbb{R}^N$*, the KNN Classifier first selects the* k *nearest training samples* $\left\{\mathbf{x}^{(1)}, \mathbf{x}^{(2)}, \ldots, \mathbf{x}^{(k)}\right\}$ *under measure* D*, and subsequently calculates the mode of* $\left(y^{(1)}, y^{(2)}, \ldots, y^{(k)}\right)$ *, where* $y^{(i)}$ *denotes the ground-truth integer label of sample* $\mathbf{x}^{(i)}, i \in \{1, \ldots, k\}$ *, as the predicted label of* $\mathbf{x}^{(0)}$*.*

LTD-only variant of the Purkinje-Nuclei Classifier

Based on classic theory alone, we propose a minimal setup that enables multi-class classification. Let $\mathbf{y}, \hat{\mathbf{y}} \in \mathbb{R}^q$ denote the true and predicted one-hot encoded labels for a single sample, respectively. In the IO, the error signals are computed as:

$$\mathbf{e} = \mathrm{ReLU}(\mathbf{y} - \hat{\mathbf{y}}) \ , \tag{S29}$$

where Rectified Linear Unit (ReLU) is applied as the activation function of IO neurons. In the scenario of incorrect prediction, $\mathbf{e} = \mathbf{y}$, with non-zero entry corresponding to the FN error. The error signals are transmitted to PCs via CFs, where LTD is applied alone, decreasing the weights from GrCs to PCs when a presynaptic activation coincides with a complex spike:

$$\mathbf{W} \leftarrow \mathbf{W} - \alpha\, \mathbf{g}^\top \mathbf{e} \ , \tag{S30}$$

where $\mathbf{g}$ is the GrC code and α is the learning rate. Specifically, only the weights of the PC corresponding to the FN error position are decreased, thereby disinhibiting its downstream CN for correct responses.

Theoretically, the LTD-only variant could only correct FN errors, intuitively leading to systematic inefficiency in supervised learning. To investigate this, we trained and evaluated an LTD-only variant on the fMNIST task with a per-class accuracy breakdown. The GrC coding process was identical to Fig 4B in the main text. The Purkinje-Nuclei Classifier and the LTD-only variant were separately trained utilizing GrC codes as input, with batch size 200 for 100 epochs. Aligning with the prediction, in each class, the training and test accuracy of the LTD-only variant were substantially lower than the Purkinje-Nuclei Classifier with both LTD and LTP (S2 Fig). Additionally, the overall training (80.01%) and test accuracy (77.69%) of the LTD-only variant were significantly lower.

Activity-dependent bias adjustment

We will demonstrate that the activity-dependent bias adjustment addresses the dead-neuron problem inherent in sparse coding schemes and facilitates the decorrelation of GrC firing patterns.

Because of the translation invariance of the top-k operation, the bias update algorithm, i.e., Eq.(5) in the main text, is equivalently expressed without predefining an expected firing rate:

$$\mathbf{b}_j \leftarrow \mathbf{b}_j - \eta\, \mathbf{f}_j, \quad j = 1, \ldots, N, \tag{S31}$$

where $\mathbf{b}_j$ and $\mathbf{f}_j$ represent the bias term and firing rate in P samples, respectively, for the j-th GrC among total N GrCs, and η denotes the learning rate. This reveals that the bias adjustment would

converge to the state where $\mathbf{f}_j \equiv \mu, \forall j \in \{1, \ldots, N\}$, with μ representing a constant firing rate for all GrCs. Upon reaching convergence, while the bias term adjustment continues, the GrC codes stabilize. Consider a system with n bins, each containing l GrCs, yielding a total population of $N = n \times l$ GrCs. Let $\mathbf{G} \in \mathbb{R}^{P \times N}$ represent the GrC code matrix. Given the fixed number of winners k in each bin, the non-zero entries in $\mathbf{G}$ could be calculated:

$$
\begin{aligned}
& (\mu P)\, N = (kn)\, P \\
\iff\ & \mu = \frac{kn}{N} = \frac{kn}{nl} \\
\iff\ & \mu = \frac{k}{l}\ .
\end{aligned}
\tag{S32}
$$

Consequently, $\frac{k}{l}$, i.e., the sparsity value, is the expected firing rate to be converged for each GrC. In our implementation, we set the sparsity between 1% and 10%, ensuring that each GrC activates in only a small fraction of samples after the bias adjustment.

Efficient GrC code processing algorithms

By leveraging properties of sparse binary GrC codes and the established theorems, we have developed efficient algorithms for unsupervised activity-dependent bias adjustment and supervised training of the Purkinje-Nuclei Classifier. Let $\mathbf{G} \in \mathbb{R}^{P \times N}$ denote the GrC code matrix, where P represents the total number of samples and N the total number of GrCs. Let $k \in \mathbb{Z}^+$ denote the number of selected winners among the N GrCs.

For the GrC codes storage, we utilize a winner-indexing matrix $\mathbf{C} \in \mathbb{R}^{P \times k}$, where each row stores the indices of the winning GrCs for the corresponding sample. Let $\mathbf{0} \in \mathbb{R}^N$ denote the N-dimensional zero vector. For $i \in \{1, \ldots, P\}$, $\mathbf{0}$ is transformed into $\mathbf{G}_{i,\cdot}$ by setting the entries at positions $\mathbf{C}_{i,\cdot}$ to 1. Notably, this can equivalently be interpreted as counting the occurrence frequencies of $\{1, \ldots, N\}$ in $\mathbf{C}_{i,\cdot}$, as each GrC fires at most once per sample.

As demonstrated in Eq. (S31), the core computational requirement for the activity-dependent bias adjustment is calculating the firing rates of all GrCs, represented by the vector $\mathbf{f} \in \mathbb{R}^N$. This is achieved by continuously counting frequencies through all samples. The matrix $\mathbf{C}$ is first flattened into a vector $\overset{vec}{\mathbf{C}} \in \mathbb{R}^{(P \times k)}$ by concatenating rows. The frequencies of $\{1, \ldots, N\}$ in $\overset{vec}{\mathbf{C}}$ are subsequently counted and divided by P to obtain $\mathbf{f}$.

For the Purkinje-Nuclei Classifier, we present two efficient computational implementations. The first computes the PCs output $\mathbf{Z}^{(r)}_{i,\cdot} = (\mathbf{G}_{i,\cdot})\, \mathbf{W}^{(r)}, i \in \{1, \ldots, P\}$ through the summation of rows in $\mathbf{W}^{(r)}$:

$$
\mathbf{Z}^{(r)}_{i,\cdot} = \sum_{j=1}^{k} \mathbf{W}^{(r)}_{(\mathbf{C}_{i,j},\cdot)}\ ,\ \ i \in \{1, \ldots, P\}\ . \tag{S33}
$$

The second implementation efficiently computes the weights adjustment $\Delta\mathbf{W}^{(r+1)} = \mathbf{G}^\top \left(\hat{\mathbf{Y}}^{(r)} - \mathbf{Y}\right)$. Let $\mathbf{E}^{(r)} = \hat{\mathbf{Y}}^{(r)} - \mathbf{Y}$ for clarity. We have:

$$\left(\Delta\mathbf{W}_{\cdot,j}^{(r+1)}\right)^\top = \left(\mathbf{E}_{\cdot,j}^{(r)}\right)^\top \mathbf{G} \ , \ j \in \{1, \dots, q\} \ . \tag{S34}$$

Note that each entry of $\mathbf{E}_{\cdot,j}^{(r)}$ is restricted to $\{-1, 0, 1\}$. Let $\mathcal{E}^{(r),j,-1}$ and $\mathcal{E}^{(r),j,+1}$ represent the vectors of indices for the -1 entries and $+1$ entries in $\mathbf{E}_{\cdot,j}^{(r)}$, $j \in \{1, \dots, q\}$. We extract the corresponding submatrices of $\mathbf{C}$, i.e., $\mathbf{C}_{\left(\mathcal{E}^{(r),j,-1},\cdot\right)}$ and $\mathbf{C}_{\left(\mathcal{E}^{(r),j,+1},\cdot\right)}$, subsequently flattening them into vectors $\overset{vec}{\mathbf{C}}_{\left(\mathcal{E}^{(r),j,-1},\cdot\right)}$ and $\overset{vec}{\mathbf{C}}_{\left(\mathcal{E}^{(r),j,+1},\cdot\right)}$. Counting the frequencies of $\{1, \dots, N\}$ in respective flattened vectors generates $\mathbf{Counts}^{(r),j,-1}$ and $\mathbf{Counts}^{(r),j,+1}$. We have:

$$\left(\Delta\mathbf{W}_{\cdot,j}^{(r+1)}\right)^\top = \mathbf{Counts}^{(r),j,+1} \ - \ \mathbf{Counts}^{(r),j,-1} \ , \ j \in \{1, \dots, q\} \ . \tag{S35}$$

Dataset-dependent generalization performance on visual tasks

In the supervised learning framework, a model is directly fitted to the training dataset, with its internal representations reflecting the distribution of the training data. If the test set samples could not be captured by the training set distribution, the internal representations of the model might fail in generalizing to these test samples. To further investigate this, we implemented a reshuffle procedure informed by the Uniform Manifold Approximation and Projection (UMAP) visualization of the fMNIST dataset. The UMAP of the raw fMNIST dataset revealed that certain test samples exhibited large deviations from the training distribution, increasing their likelihood of misclassification (S6A Fig). Therefore, we reshuffled the training and test set assignments. Within each class, we selected approximately 2000 samples that form local clusters in the UMAP, subsequently randomly assigning 1000 of them to the test set, resulting in total $10,000$ (identical number to original) test set samples across all classes, with the remainder defined as the training set. The GrC coding process was identical to Fig 4B in the main text, and the Purkinje-Nuclei Classifier was subsequently trained with batch size 200 for 100 epochs. From our KNN-based interpretation of GrC representations, the reshuffled dataset was expected to yield substantially improved performance, as the newly constructed test set maintained local cluster distribution captured in the training distribution (S6B Fig). In line with this prediction, the model achieved a training accuracy of 100.00% and a test accuracy of 96.79% on the reshuffled dataset, demonstrating that reshuffled sample distribution significantly enhanced the generalization capability (S6C Fig). However, we underscore that this result is presented solely to illustrate the impact of training-test data distribution, as is common practice to maintain the standard dataset split for the fMNIST benchmark.

Furthermore, as **Theorem 5** proves that our model achieves perfect training accuracy under certain conditions, it is straightforward to consider increasing the number of training samples to more accurately

approximate the true underlying data distribution and improve the test accuracy. To investigate this, we randomly split the entire $70,000$ samples of the fMNIST dataset, systematically varying the training-set size. For each configuration of training-set size (500, 1000, 2000, 5000, $10,000$, $20,000$, $30,000$, $40,000$, $50,000$, $60,000$, $65,000$, $68,000$), the GrC coding process was identical to Fig 4B in the main text, and the Purkinje-Nuclei Classifier was subsequently trained with batch size of 200 for 30 epochs. The result demonstrated that our model retained a moderate generalization capability (test accuracy $81.96 \pm 0.34\%$) when trained on merely 1000 samples and tested on the remaining $69,000$. Furthermore, the test accuracy exhibited a gradual increase with larger training-set size, confirming that the generalization performance is dependent on the quantity of training data (S7 Fig).

Additionally, we trained and evaluated the model on the visual classification benchmark dataset MNIST, which consists of $60,000$ training and $10,000$ test grayscale images of size 28×28 across 10 categories of handwritten digits, reporting the mean $\pm$ standard deviation of 10 random seeds. Note that 32-bit float/integer was used for assessing computation speed in additional experiments. Following each random initialization, the mossy fiber sampling used local RF of 4×4, exhaustively applied to all possible sliding windows, yielding $(29 - 4)^2 = 625$ bins. Each bin contained 24 GrCs with 1 winner selected, yielding a total of $15,000$ GrCs. The Purkinje-Nuclei Classifier was subsequently trained with batch size 200 for 50 epochs. Our model achieved a test accuracy of $97.59\% \pm 0.05\%$, comparable to the single-hidden-layer artificial neural network (test accuracy 96.380%) reported in the previous study [1]. The accuracy gap between training and test on the MNIST task is substantially lower than the fMNIST, illustrating that our model could extend to other visual dataset with significantly enhanced generalization capability.

Real-time continuous learning approach in motor tasks

In pursuit of rapid computations in motor tasks, the entire observation dataset was first sampled and subsequently used to train the model, as described in the main text. Alternatively, our model could be trained simultaneously with data collection using real-time continuous simulations in the environment, illustrating an approach of increased biological plausibility.

The real-time continuous learning approach consists of n-step simulation-and-training process, which is iterated for certain duration starting from $t = 0$. In each cycle of n-step process, the real-time simulation is first conducted with the cerebellar model interacting with the environment for n steps. The heuristic strategy S, as defined in the Methods section, is subsequently utilized to determine whether cerebellar actions in these n steps are optimal, with training applied retrospectively at steps where the cerebellar action differs from the heuristic optimal action (S10A Fig). This procedure is conceptually analogous to our original approach of static supervised learning with batch size n, except that these n training samples are generated through continuous interaction with the environment. We investigated the 1-step and 2-step cases, compared with our original approach in the CartPole task. Note that 32-bit

float/integer was used in the following experiment.

In the original approach, $200,000$ observation-action pairs were first sampled using the heuristic strategy S. Differing from the main text, the whole sampled dataset was defined as the training set. The GrC coding process used identical hyperparameters as specified in the main text, except for the activity-dependent bias adjustment, which was applied with learning rate 10^{-4} and batch size 5 for 5 epochs. The Purkinje-Nuclei Classifier was subsequently trained using batch size 5 for 3 epochs (corresponding to $600,000$ timesteps in total), with evaluation on $10,000$ randomly initialized trials at selected training durations (corresponding to 100, 500, 2000, 9000, $30,000$, $50,000$, $70,000$, $100,000$, $600,000$ timesteps). The 1-step and 2-step cases used identical granular-layer hyperparameters as the original approach, except for the activity-dependent bias adjustment, which was not applicable to the 1-step case without firing rate, and was applied with learning rate 10^{-4} and batch size 2 for each 2-step simulation-and-training process. The Purkinje-Nuclei Classifier was continuously trained for each n-step process as described above. The evaluation procedure of the 1-step and 2-step cases was identical to the original approach.

The evaluation result demonstrated that the real-time continuous learning approach could be feasible, but learned substantially more slowly than the original approach (S10B Fig). Specifically, the original approach first reached the successful performance criterion of 500 evaluation timesteps after approximately 9000 training timesteps. By contrast, the real-time continuous learning approach exhibited slower learning curves. The 1-step case did not reach the 500-timestep success criterion even after $600,000$ training timesteps, whereas the 2-step case reached this criterion after approximately $50,000$ training timesteps. Therefore, our model could learn through continuous interaction with the environment and eventually achieve comparable performance to the original approach, while the original approach could provide substantially faster learning speed.

WTA implementation by overlapping GoC inhibitory fields

In this section, we propose further computational implementation of WTA in the granular layer. Based on the anatomical and physiological evidence in the literature, we define a bin as a spatially localized group of GrCs that share identical sampled mossy fiber inputs, within which the localized WTA process is attributed to GoC-mediated feedback inhibition. In the main text, the top-k operation directly selects the k largest entries within each bin as a simplistic implementation of the WTA process. Interestingly, while the actual GoC-GrC connectivity is complex, directly selecting the most-active GrCs with the top-k operation could be functionally similar to implementing the localized WTA through overlapping GoC inhibitory fields under certain conditions, as demonstrated below.

Consider a system comprising n bins, each containing l GrCs. For a given raw input, let $\mathbf{x}^{(i)} \in \mathbb{R}^m$ denote the i-th sampled mossy-fiber value, which serves as the input to the i-th bin after normalization by its Euclidean norm. Let $\mathbf{T}^{(i)} \in \mathbb{R}^{m \times l}$ denote the MF-GrC weights for the i-th bin, where each weight

vector of GrC is normalized by its Euclidean norm. As defined in the Methods section, the GrCs value of the i-th bin before the WTA process is denoted by $\mathbf{s}^{(i)} \in \mathbb{R}^l$:

$$\mathbf{s}^{(i)} = \mathbf{x}^{(i)}\mathbf{T}^{(i)} \quad . \tag{S36}$$

In the original top-k implementation, the k most-active GrCs are directly selected as winners within each bin, i.e., setting the k largest entries of $\mathbf{s}^{(i)}$ to 1 and the remaining entries to 0.

Alternatively, in the case of overlapping GoC inhibition, consider the instance where a GoC receives input from a subset of GrCs in the i-th bin (without loss of generality, consider the 1st to the $p^{(i)}$-th GrC, where $1 \leq p^{(i)} \leq l$) and the j-th bin (1st to the $p^{(j)}$-th GrC), and provides feedback inhibition with strength λ to these GrCs. The inhibition value Inh is calculated as:

$$Inh = \lambda \left(\sum_{k=1}^{p^{(i)}} \mathbf{s}_k^{(i)} + \sum_{k=1}^{p^{(j)}} \mathbf{s}_k^{(j)} \right) \quad . \tag{S37}$$

The WTA process could be modeled as filtering through the threshold Inh with the Heaviside function, yielding the sparse GrC representation $\mathbf{g}^{(i)} \in \mathbb{R}^l$ for the i-th bin:

$$\mathbf{g}^{(i)} = \text{Heaviside}\left(\mathbf{s}^{(i)} - Inh\right) \quad . \tag{S38}$$

Crucially, selecting the k largest entries in $\mathbf{s}^{(i)}$ would be functionally equivalent to filtering through a certain threshold Inh with the Heaviside function, which sets values smaller than Inh to 0 and values larger than Inh to 1. Therefore, provided that weights in the GoC-GrC circuit remain constant, we would propose a perspective that, in terms of sparse GrC coding, the non-overlapping top-k implementation would presumably approximate the functional outcome of overlapping GoC inhibition.

To further validate this functional approximation, we constructed cerebellar models with the granular layer featuring overlapping GoC inhibitory fields (S11A Fig). Cases of varying overlapping degree d were tested and compared with our original top-k implementation on the MNIST task, with the mean $\pm$ standard deviation across 10 randomly initialized trials reported. Note that MNIST was used as a computationally tractable benchmark for testing whether the coding statistics and downstream accuracy could be preserved under the overlapping inhibition case, intended to validate the functional approximation of the top-k implementation rather than to report optimal performance on the MNIST benchmark. The mossy fiber sampling used local 4×4 RF, exhaustively applied to all possible sliding windows, resulting in 625 bins with each containing 24 GrCs. For the top-k implementation, 1 winner was directly selected in each bin. For the overlapping inhibition cases, the WTA was implemented as per Eqs. (S36) to (S38). Note that $d = 0$ corresponds to the non-overlapping case implemented with GoC inhibition value calculated by Eqs. (S36) to (S38), which is different from the original top-k implementation. Following the WTA, all cases implemented the Purkinje-Nuclei Classifier trained with batch size 200 for 50 epochs. Note that the maximal test accuracy in 50 epochs was recorded as the final metric for each randomly initialized trial.

Given the inhibition strength λ appropriately tuned, an overlapping inhibition model of arbitrary d value would be expected to produce similar sparse coding properties and task performance to the original model, while the original top-k implementation would offer significant computational acceleration. The numerical result aligned with this theoretical prediction, as the test accuracy displayed no statistically significant difference (S11C Fig), and the Python script execution time of the top-k implementation was significantly faster (S11D Fig), irrespective of d value. In addition, the GrC firing distribution of the top-k implementation closely resembled that of the overlapping inhibition cases (S11B Fig). Therefore, the non-overlapping top-k simplification in our original implementation could inherently promote sparsity among GrCs and effectively capture essential GrC coding properties, with substantial computational acceleration provided.

Cerebellum-inspired dynamic spiking framework

The primary objective of our study is to develop a cerebellum-inspired, lightweight neural network for rapid supervised learning of input-output mapping. Nevertheless, the computational principles from our static, rate-based model could be extended to a dynamic spiking framework while maintaining comparable accuracy on the classification task, as demonstrated in the following proof-of-principle experiment.

In the dynamic spiking framework, the PC is built as a quadratic leaky integrate-and-fire model, governed by Eqs. (S39) to (S46) with tuned parameters that capture the nature of simple and complex spikes of the PC [2].

$$g_{\mathrm{pf}}(t) = \sum_{k=1}^{N} \left\{ g_k \left[e^{-\left(\frac{t-t_k}{1.2}\right)} - e^{-\left(\frac{t-t_k}{0.5}\right)} \right] \mathrm{Heaviside}\left(t - t_k\right) \right\} \quad , \tag{S39}$$

$$g_{\mathrm{cf}}(t) = 3.93 \left[e^{-\left(\frac{t-t_{\mathrm{cf}}}{8.58}\right)} - e^{-\left(\frac{t-t_{\mathrm{cf}}}{0.29}\right)} \right] \mathrm{Heaviside}\left(t - t_{\mathrm{cf}}\right) \quad , \tag{S40}$$

$$g_{\mathrm{syn}}(t) = g_{\mathrm{pf}}(t) + g_{\mathrm{cf}}(t) \quad , \tag{S41}$$

$$I_{\mathrm{syn}}(t) = g_{\mathrm{syn}}(t)\left(v - E_{\mathrm{exc}}\right) \quad , \tag{S42}$$

$$\frac{\mathrm{d}v}{\mathrm{d}t} = 0.04v^2 + 5v + 144.68 - u + 30.68p - I_{\mathrm{syn}}(t) \quad , \tag{S43}$$

$$\frac{\mathrm{d}u}{\mathrm{d}t} = 0.19(0.22v - u) \quad , \tag{S44}$$

$$\frac{\mathrm{d}p}{\mathrm{d}t} = -\frac{p}{11.17} + 1.04\,(1-p)\,e^{-\left(\frac{t-t_{\mathrm{cf}}}{2.75}\right)}\mathrm{Heaviside}\,(t - t_{\mathrm{cf}}) \quad , \tag{S45}$$

$$\begin{cases} v \leftarrow -74.46, \\ u \leftarrow u + 15 + 3p, \end{cases} \quad \text{if } v \geq 30\,\mathrm{mV} \ . \tag{S46}$$

The parallel fiber conductance $g_{\mathrm{pf}}(t)$ arises from presynaptic GrC inputs, where g_k is the peak conductance (synaptic weight) for the k-th synapse, and t_k is the arrival time of its incoming spike. The total synaptic conductance $g_{\mathrm{syn}}(t)$ is obtained as the sum of the parallel fiber conductance $g_{\mathrm{pf}}(t)$ and the climbing fiber conductance $g_{\mathrm{cf}}(t)$. $I_{\mathrm{syn}}(t)$ is the conductance-based excitatory postsynaptic current (EPSC) governed by Eqs. (S39) to (S42). E_{exc} represents the reversal potential of the excitatory synapse, set to 0 mV. v is the membrane potential, u is the adaptation variable, and p is the plateau variable triggered by the climbing fiber input, with t_{cf} denoting the timing of the climbing fiber input. In the spontaneous state (i.e., $I_{\mathrm{syn}}(t) = 0$), the PC model exhibited a firing pattern of simple spikes with a firing rate of approximately 60 Hz.

Based on these equations, we modified our original rate-based model of PC (S12A Fig) to a dynamic spiking version (S12B Fig). Consider a system comprising N GrCs and q PCs. In the original model, the PCs value $\mathbf{z} \in \mathbb{R}^q$ is computed via linear integration $\mathbf{z} = \mathbf{x}\mathbf{W}$, where $\mathbf{x} \in \{0,1\}^N$ is the GrC code, and $\mathbf{W} \in \mathbb{R}^{N\times q}$ denotes the GrC-PC synaptic weights. In the spiking implementation, each PC is instantiated as a spiking neuron governed by the dynamics described from Eqs. (S39) to (S46), with the binary GrC code converted into presynaptic spiking events delivered to postsynaptic PCs. Specifically, each active GrC emits one spike, with its arrival time randomly assigned within a 50-ms simulation window, and the GrC-PC weight matrix $\mathbf{W}$ determines the peak conductance of the evoked postsynaptic current. Under this setting, the membrane potential v of each PC evolves dynamically, with the total number of output spikes accumulated over the 50-ms simulation window counted for downstream classification. The update of the weight matrix $\mathbf{W}$ utilizes the identical learning rule as the original rate-based model, namely the Multi-Class Perceptron algorithm. Note that the learning only involves simple spikes, while CF-evoked complex spikes are modeled but not yet incorporated.

As a proof-of-concept illustration, we trained and evaluated the spiking model on a binary classification case with two categories ("T-shirt" and "ankle boot") selected from the original fMNIST dataset. Theoretically, the spiking simulation would be significantly more computationally demanding than the original model, primarily attributed to the simulation of spiking dynamics, which requires recurrent computations over a time window. For instance, given the time step $\mathrm{d}t = 0.01$ ms and 50-ms window, this entails a large number of 5000 integration steps per forward pass. In line with this prediction, our original model was more than 10^3 times faster than the spiking model (S12D Fig), despite the

well-optimized implementation of the spiking model for acceleration. Nevertheless, the spiking model successfully achieved a test accuracy comparable to that of our original model utilizing the same GrC codes (S12C Fig). The two PCs exhibited distinct firing counts in response to given inputs (S12E Fig), thereby successfully differentiating between two categories. Therefore, under the proposed theoretical framework of supervised learning, the extension to a more biologically plausible spiking model could be a feasible and highly promising research direction.

Cerebellar Model for Regression

Definition 7 (Cerebellar Model for Regression). *Consider N-dimensional GrC codes and q-dimensional target values of total P samples, represented by matrices $\mathbf{G} \in \mathbb{R}^{P\times N}$ and $\mathbf{Y} \in \mathbb{R}^{P\times q}$, respectively, with each row corresponding to a single sample. Let $\mathbf{W}^{(r)} \in \mathbb{R}^{N\times q}$ denote the weight matrix from the input layer to the output layer after r epochs of training. Let $\mathbf{J} \in \mathbb{R}^{N\times q}$ denote the all-ones matrix of dimension $N \times q$, and $\lambda \in \mathbb{R}$ represents a sufficiently large positive constant. After r epochs, the linear integration in PCs is calculated as $\mathbf{Z}^{(r)} \in \mathbb{R}^{P\times q}$:*

$$\mathbf{Z}^{(r)}_{i,\cdot} = (\mathbf{G}_{i,\cdot})\left(\mathbf{W}^{(r)} - \lambda\mathbf{J}\right) \quad , \quad i \in \{1, \dots, P\} \quad . \tag{S47}$$

The final CNs output is $-\mathbf{Z}^{(r)}$, as predictions of target values. The loss value after r epochs is calculated as:

$$L^{(r)}_{\mathrm{SSE}} = \frac{1}{2}\sum_{i=1}^{P}\left\|\mathbf{Y}_{i,\cdot} + \mathbf{Z}^{(r)}_{i,\cdot}\right\|_2^2 \quad . \tag{S48}$$

Applying gradient descent optimization, the weight correction applied at the r-th epoch is computed as:

$$\Delta\mathbf{W}^{(r)}_{\mathrm{SSE}} = -\alpha\,\mathbf{G}^{\top}\left(\mathbf{Y} + \mathbf{Z}^{(r-1)}\right) \quad , \tag{S49}$$

where α is the learning rate. This can be rewritten in the following form of update algorithm:

$$\begin{aligned} &\text{for } i \in \{1, \dots, P\} : \\ &\quad \mathbf{W}^{(r)} = \mathbf{W}^{(r-1)} - \alpha\,(\mathbf{G}_{i,\cdot})^{\top}\left(\mathbf{Y}_{i,\cdot} + \mathbf{Z}^{(r-1)}_{i,\cdot}\right) \quad . \end{aligned} \tag{S50}$$

Provided that $\mathbf{G}^{\top}\mathbf{G}$ is invertible, solving the normal equation gives:

$$\mathbf{W}^{(+\infty)} = \lambda\mathbf{J} - \left(\mathbf{G}^{\top}\mathbf{G}\right)^{-1}\mathbf{G}^{\top}\mathbf{Y} \; . \tag{S51}$$

Our study primarily focuses on the CF-error-driven multi-class classification. However, provided that CFs could transmit graded signals, we have employed minor modifications to adapt the cerebellar model for regression tasks. The GrC coding process remains unchanged, and GrC codes continue to serve as

inputs to the Purkinje layer. By introducing an inhibitory interneuron that sums all GrC inputs and projects inhibitory connections to all PCs with fixed weight of λ, we derive Eq. (S47), which defines the PCs output $\mathbf{Z}^{(r)}$. Due to the inhibitory nature of PCs, the final CNs output is $-\mathbf{Z}^{(r)}$, which is transmitted to the IO to compute the error signals $\mathbf{E} = \mathbf{Y} - \left(-\mathbf{Z}^{(r)}\right) = \mathbf{Y} + \mathbf{Z}^{(r)}$. For each sample, the continuous version of synaptic plasticity rule is modeled such that weight decreases are proportional to pre-synaptic GrC activations and post-synaptic CF error signals, as demonstrated in Eq. (S50). Given a sufficiently large sample size and a small number of GrCs, the $\lambda\mathbf{J}$ term in Eq. (S51) justifies the inclusion of the additional inhibitory interneuron in the Purkinje layer, as $\mathbf{W}$ cannot assume negative values in biologically plausible scenarios.

We evaluated the performance of the Cerebellar Model for Regression on the California Housing Dataset imported from scikit-learn, compared with an FNN trained via backpropagation using the Adam optimizer (S13 Fig). This dataset contains 8-dimensional inputs and 1-dimensional target values for total $20,640$ samples, and was partitioned into training and test sets with a sample ratio of $8:2$. For the cerebellar model, each RF comprised 2 components from the 8-dimensional observation vector, generating $C_8^2 = 28$ bins, each populated by 350 GrCs, resulting in a total of 9800 GrCs. The top 5% of GrCs in each bin were selected as winners, and the activity-dependent bias adjustment was applied with learning rate 6×10^{-4} and batch size 100 for 100 epochs. Subsequently, the Purkinje layer was trained for regression with learning rate 5×10^{-6} and batch size 1651 for 550 epochs, achieving an $R^2 = 0.82$ for the test set. The FNN contained 9800 hidden units and was trained with learning rate 3×10^{-4} and batch size 1651 for $20,000$ epochs, achieving an $R^2 = 0.77$ for the test set.

References

1. Beohar D, Rasool A. Handwritten Digit Recognition of MNIST dataset using Deep Learning state-of-the-art Artificial Neural Network (ANN) and Convolutional Neural Network (CNN). In: 2021 International Conference on Emerging Smart Computing and Informatics (ESCI); 2021. p. 542-8. doi:10.1109/ESCI50559.2021.9396870.

2. Zang Y, Dieudonné S, Schutter ED. Voltage- and Branch-Specific Climbing Fiber Responses in Purkinje Cells. Cell Reports. 2018;24:1536-49. doi:10.1016/j.celrep.2018.07.011.

Table A. Ablation studies for the cerebellar model on the fMNIST task. This ablation study demonstrates that the receptive field design, bin architecture, and unsupervised bias adjustment constitute critical components contributing to the performance on fMNIST. For all four experiments, 4 mossy fibers were connected to each GrC, the top 10% of GrCs were selected as winners, and the Purkinje-Nuclei Classifier was trained using batch size 200 for 100 epochs.

**Mossy fibers**		**Granular layer**		**Activity-dependent bias**				**Purkinje-Nuclei Classifier**
**sampling**	**architecture**	**GrCs**	**winners**	**epoch**	**batch size**	**learning rate**	**dead neuron**	**test accuracy**
random	classic	$50,000$	5000	/	/	/	0.206%	87.34%
random	bin	2500 bins $\times$ 20	2 in a bin	/	/	/	1.428%	89.68%
combined	bin	2500 bins $\times$ 20	2 in a bin	/	/	/	1.132%	90.42%
combined	bin	2500 bins $\times$ 20	2 in a bin	5	100	10^{-3}	0.046%	90.59%
